# Circadian Clock Model Supports Molecular Link Between *PER3* and Human Anxiety

**DOI:** 10.1038/s41598-017-07957-4

**Published:** 2017-08-31

**Authors:** Amanda R. Liberman, Soo Bin Kwon, Ha T. Vu, Allan Filipowicz, Ahmet Ay, Krista K. Ingram

**Affiliations:** 1 Biology Department, Colgate University, Hamilton, NY 13346 USA; 2 Bioinformatics Program, University of California, Los Angeles, CA 90024 USA; 3 Mathematics Department, Colgate University, Hamilton, NY 13346 USA; 4 Johnson Graduate School of Management, Cornell University, Ithaca, NY 14850 USA

## Abstract

Generalized anxiety and major depression have become increasingly common in the United States, affecting 18.6 percent of the adult population. Mood disorders can be debilitating, and are often correlated with poor general health, life dissatisfaction, and the need for disability benefits due to inability to work. Recent evidence suggests that some mood disorders have a circadian component, and disruptions in circadian rhythms may even trigger the development of these disorders. However, the molecular mechanisms of this interaction are not well understood. Polymorphisms in a circadian clock-related gene, *PER3*, are associated with behavioral phenotypes (extreme diurnal preference in arousal and activity) and sleep/mood disorders, including seasonal affective disorder (SAD). Here we show that two *PER3* mutations, a variable number tandem repeat (VNTR) allele and a single-nucleotide polymorphism (SNP), are associated with diurnal preference and higher Trait-Anxiety scores, supporting a role for *PER3* in mood modulation. In addition, we explore a potential mechanism for how *PER3* influences mood by utilizing a comprehensive circadian clock model that accurately predicts the changes in circadian period evident in knock-out phenotypes and individuals with *PER3*-related clock disorders.

## Introduction

The circadian clock controls the oscillation of an approximately 24-hour sleep-wake cycle. Gene mutations that disrupt this molecular pacemaker influence sleep and activity timing and are associated with numerous behavioral and health disorders, including depression and mental health syndromes, obesity, coronary disease, and cancer^1–9^. In this study, we examine one clock gene, *period homolog 3* (*PER3*). *PER3* is often implicated in genome-wide association studies of mood and sleep-related disorders^10–12^, yet the role of *PER3* in regulating circadian rhythms is still unclear. While *PER1* and *PER2* are considered to be part of the core mammalian circadian clock network^13^, *PER3* is thought to be non-essential^14^. Mice deficient in *Per3* have functioning circadian clocks, but these mice express a key phenotypic difference–a circadian period that is shorter than in wild-type mice^14^. Due to the uncertainty of the role of *PER3* in regulating circadian rhythms, previous mathematical models of the circadian clock have neglected to include *PER3* as a distinct component of the circadian clock network^15–22^.

Despite the limited role of *PER3* in the central clock mechanism, multiple SNPs and VNTRs in *PER3* have been linked to diurnal preference^23^, or chronotype, the circadian phenotype that describes an individual’s peak state of arousal and preference for activity at particular times of day^11, 24–27^. Behavioral chronotypes are linked to molecular circadian rhythms via differences in the endogeneous period of the clock oscillations; evening types typically have longer periods and delayed phase shifts, and morning types have shorter periods and advanced phase shifts^28, 29^. Thus, clock gene polymorphisms associated with diurnal preference are likely to alter both the period and phase of the clock oscillation. For example, single point mutations in *PER3* are linked to familial advanced sleep phase disorder (FASPD) and delayed sleep phase disorder (DSPD)^11, 24, 25, 30, 31^. Individuals with FASPD typically have a short circadian period, an advanced circadian phase, and an extreme “morning” chronotype^32^. Conversely, individuals with DSPD typically have a long circadian period, a delayed circadian phase, and an extreme “evening” chronotype^32^. Associations between *PER3* genotypes and mood disorders have robust support in the literature^33–40^. However, the first causal link between *PER3* and circadian-related mood phenotypes was reported by Zhang *et al*.^30^, a study in which the authors describe a family group in which FASPD and SAD co-occur with a double mutation in the *PER3* gene. In humans, individuals with FASPD tend to exhibit depression and poor sleep quality^32^. When mice were transfected with the same two *PER3* variants, the animals also manifested symptoms of depression and poor sleep quality, suggesting a causal relationship between *PER3* mutations and mood^30^.

In studies of mood disorders in humans, depression and anxiety are tightly linked, and these two behavioral phenotypes tend to co-occur^41, 42^. If *PER3* mutations that alter circadian period length affect mood in humans, anxiety levels would likely be higher in individuals that had *PER3* variants associated with altered period lengths^28, 29^. Here, we test the relationship between two *PER3* variants and a well-documented measure of anxiety, the State Trait Anxiety Inventory (STAI)^43^, in morning-type (advanced phase, short period) versus evening-type (delayed phase, long period) individuals. We also utilize a comprehensive circadian clock model to explore and evaluate potential mechanisms connecting PER3 to human mood disorders.

## Results and Discussion

### *PER3* Mutations are Associated with Eveningness and Increased Trait Anxiety

**Figure 1:**
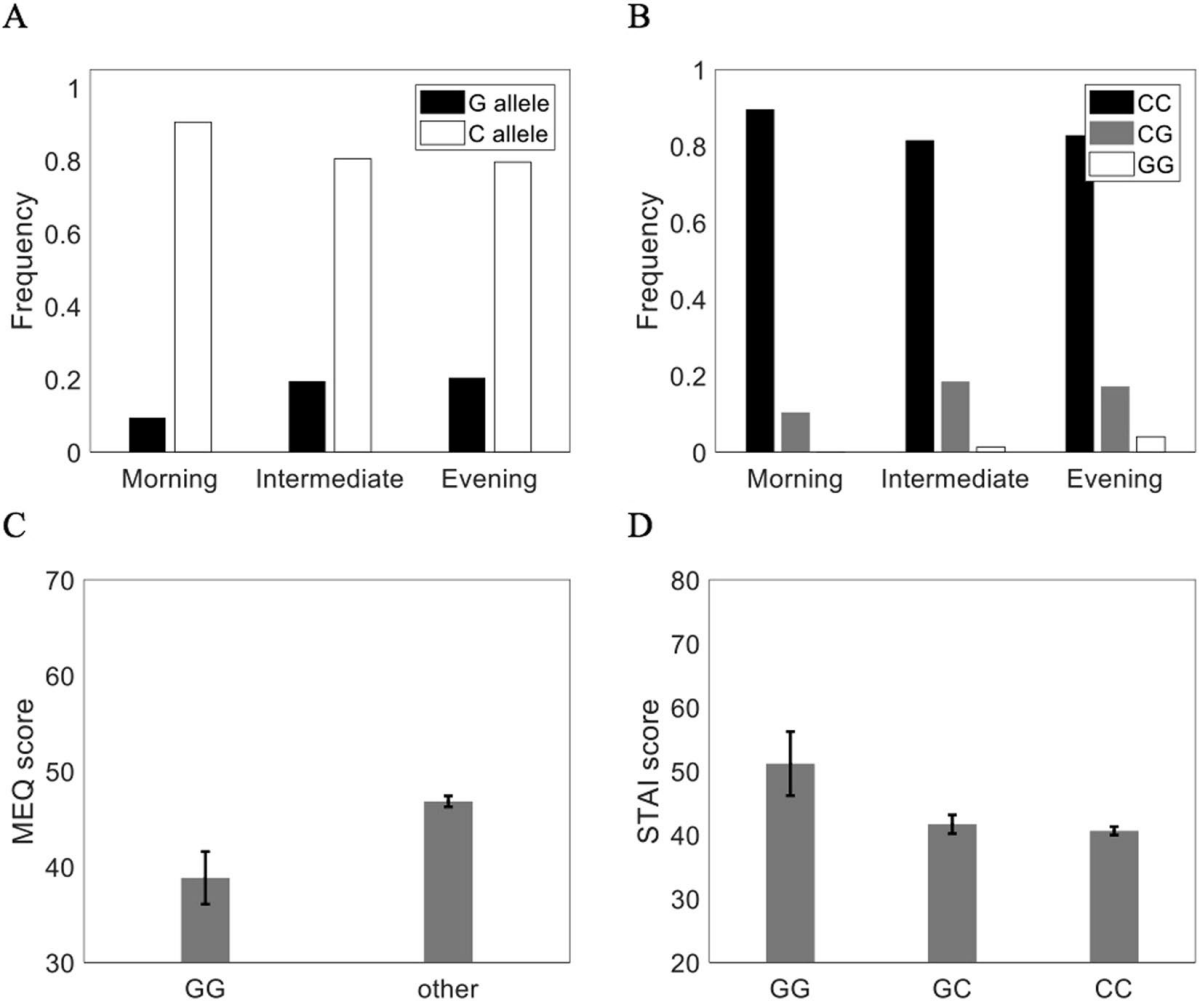
Associations of the *PER3* single nucleotide polymorphism (SNP rs228697) with circadian chronotype and anxiety. (**A**) Frequencies of C and G alleles in morning-type (n = 48), intermediates (n = 159), and evening-type (n = 103) self-reported chronotypes. The frequency of the *C* allele is lower in evening-types (OR = 17.86, 95% CI = 0.99–321.61, p = 0.05). (**B**) Frequencies of the PER3 SNP genotypes in each chronotype (χ^2^ = 29.20, *n* = 1, *p* < 0.001). Individuals homozygous for the G-allele are more likely to be evening-types. (**C**) Average self-reported MEQ scores (±SD) are lower in GG homozygotes compared to other genotypes (*t* = 7.415, *df* = 308, *p* = 0.016). (**D**) Average trait anxiety scores (±SD) are higher in GG homozygous individuals (*F*(2,305) = 3.195, *p* = 0.042).

In our study, the PER3 single nucleotide polymorphism (rs228697) is significantly associated with diurnal preference and anxiety. The frequency of the G allele is higher in evening-types (Fig. 1A; OR = 2.8, 95% CI = 0.347-22.610) and the distribution of GG homozygotes is significantly higher in extreme evening types (Fig. 1B; χ^2^ = 29.20, *n* = 1, *p* < 0.001). The average MEQ score is lower (greater propensity for eveningness) in individuals homozygous for GG than for other individuals (Fig. 1C; *t* = 7.415, *df* = 308, *p* = 0.016). In this population, individuals with at least one G allele are more anxious, on average, than other individuals (Fig. 1D; *F*(2,305) = 3.195, *p* = 0.042). Evening-types are significantly more anxious than morning-types (*M*_ET_ = 43.5, *M*_MT_ = 38.5; *t* = 8.2, *df* = 380, *p* < 0.001).

The G/C mutation at this SNP location corresponds to an amino acid substitution in exon 17 (P864A). This mutation results in a proline (P) to alanine (A) substitution and may alter the secondary structure of PER3, thus affecting kinase binding to PER3 and any function of PER3 in the regulation of circadian sleep patterns^44^. Our model, as explained below, predicts that altered binding at this site for G alleles results in a 2–6% longer circadian period (i.e., 25-hour period), supporting the prior link between period length and eveningness in a Japanese population^11^ as well as the association between eveningness, anxiety, and genotype in the present study.

**Figure 2:**
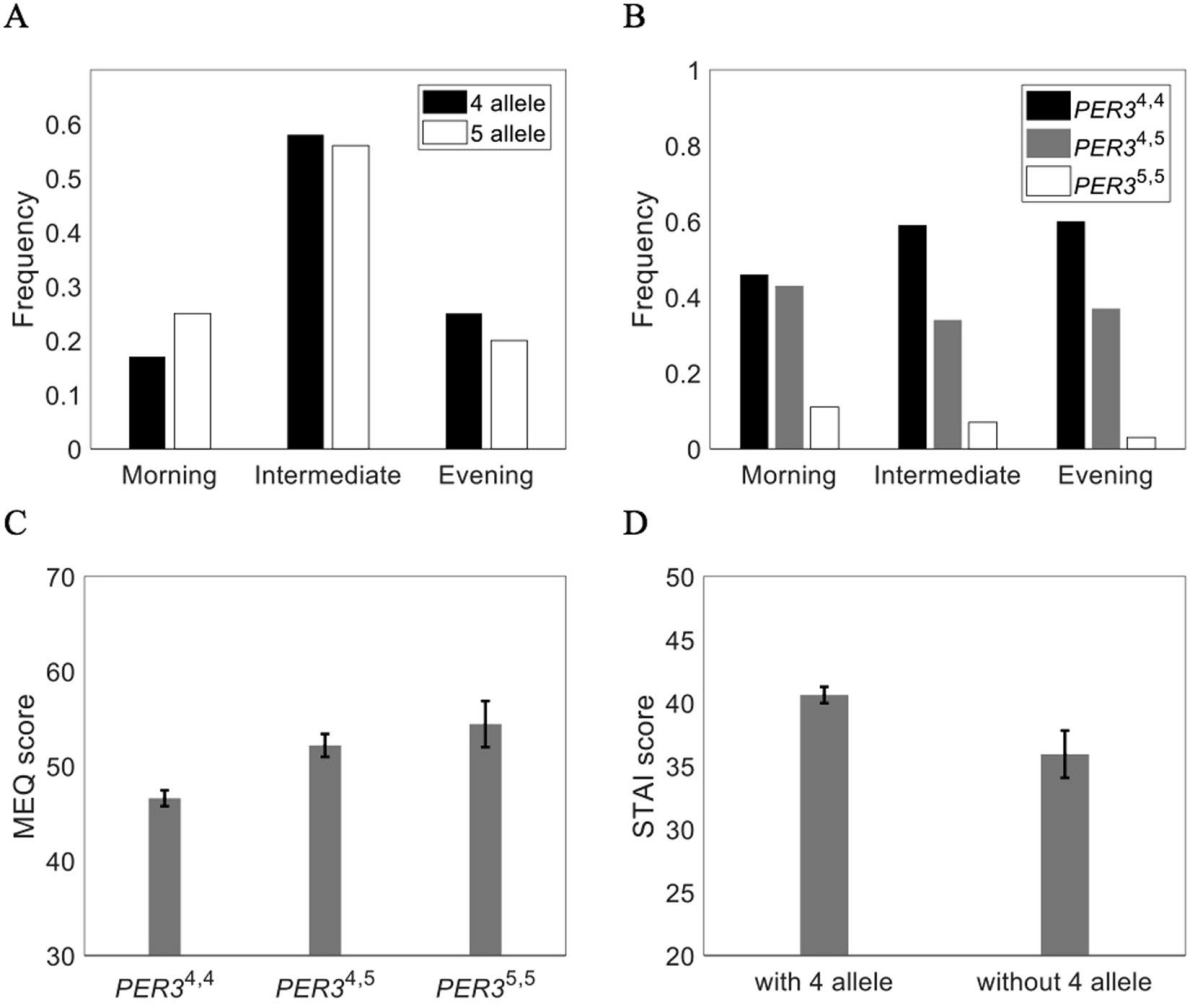
Associations of the *PER3* length polymorphism (VNTR) with circadian chronotype and anxiety. (**A**) Frequencies of 4- and 5-repeat alleles in morning-type (*n* = 46), intermediates (*n* = 136), and evening-type (*n* = 62) self-reported chronotypes. The frequency of the *PER3* ^4^ allele is higher in evening-types and lower in morning-types (OR = 0.52 95% CI = 0.280–0.975, p = 0.042). (**B**) Frequencies of the PER3 VNTR genotypes in each chronotype. *Per3* ^4,4^ homozygotes are less frequent in morning-types (χ^2^ = 5.71, *df* = 1, *p* = 0.017) and *PER3* ^5,5^ homozygotes are less frequent in evening-types (χ^2^ = 40.90, *df* = 1, *p* < 0.001). C)Average self-reported MEQ scores (±SD) are lower for *PER3* ^4,4^ homozygotes than for *PER3* ^4,5^ or *PER3* ^5,5^ types (*F(2, 242)* = 9.98, *p* < 0.001). D) Average trait anxiety scores (±SD) are higher in individuals carrying a *PER3* ^4^ allele (*t* = 2.12, *df* = 242, *p* = 0.035).

The *PER3* length polymorphism (rs57875989) is also significantly associated with diurnal preference and anxiety. The frequency of the 4-repeat allele is higher in evening-types and lower in morning-types (Fig. 2A; OR = 0.52, 95% CI = 0.280-0.975, z = 2.04, p = 0.042). *PER3* ^5,5^ homozygotes are significantly less frequent among evening-types (Fig. 2B; χ^2^ = 5.71, *df* = 1, *p* = 0.017), and *PER3* ^4,4^ homozygotes are significantly less frequent among morning types (χ^2 = ^40.9, *df* = 1, *p* < 0.001). *PER3* ^4,4^ homozygotes are five times more likely to be intermediates or evening-types than morning-types. The average MEQ score is lower (greater propensity for eveningness) in *PER3* ^4,4^ individuals than for *PER3* ^4,5^ heterozygotes or *PER3* ^5,5^ homozygotes (Fig. 2C; *F(2, 242)* = *9.98*, *p* < 0.001; post-hoc Tukey tests: *PER3* ^4,4^, *PER3* ^4,5^ p < 0.001 and *PER3* ^4,4^, *PER3* ^5,5^ p = 0.011), and individuals with the 4-repeat allele are more anxious, on average, than other individuals (Fig. 2D; *t* = 2.12, *df* = 242, *p* = 0.035).

Each of the repeat regions in the *PER3* VNTR contain potential CKIδ/ε phosphorylation sites in a tandem array. The 4-repeat allele contains fewer amino acid residues available for phosphorylation; therefore, this mutation may affect the stability and function of *PER3* via differential phosphorylation^25, 31, 44, 45^. Our model, as explained below, predicts that decreased phosphorylation results in a 2–6% longer period, supporting the previous association between the 4-repeat allele, eveningness, and DSPD^25^. Studies have also shown the reverse link between the 5-repeat allele and morningness^25, 46, 47^. Here, we have identified a new link between the 4-repeat length variant and increased anxiety, providing a potential explanation for previously reported associations between eveningness and anxiety^42, 48, 49^.

It is important to highlight the fact that *PER3* mutation effects on circadian period are bi-directional; separate mutations in the same gene are linked to phenotypes associated with both increases and decreases in circadian period lengths. From a molecular perspective, this is not surprising given that distinct mutations disrupt different functional domains of the gene. Interestingly, altering the period length in either direction results in a increase in negative affect. Thus, mood disorders appear to be generally associated with the misalignment of circadian period with light:dark cycles.

It is also important to note that mutations in other clock genes affect period length without noticeably disrupting mood^5, 50, 51^. This suggests that the effect of *PER3* in the regulation of the circadian cycle potentially alters additional downstream physiological processes that are critical for mood regulation. *PER3* is known to be involved in modulation of alerting effects of light and the influence of light on sleep^52, 53^. Thus, the differential response of *PER3* variants to light may account for the difficulty in adjusting to short periods in winter in individuals with SAD^30^ and play a role in the regulation of negative affect. The predicted links between *PER3*, circadian regulation and mood are complex and likely to involve multiple genetic pathways. As a first step, we use a modeling approach to evaluate the relative influence of key molecular interactions of *PER3* within the circadian clock network to elucidate the potential mechanisms by which *PER3* alters clock regulation.

### Human Circadian Clock Model Provides Potential Molecular Explanation for Observed *PER3*-Related Phenotypes

How does *PER3* affect circadian rhythms and mood if it is not a central component of circadian clock function? Zhang *et al*. (2016) suggested that this link may be due to decreased PER3 protein stability and reduced PER3-PER1 or PER3-PER2 binding^30^. Other potential explanations consider changes in PER3*-*CRY or CKI (kinase) binding, or the PER3 phosphorylation rate^11, 25, 44^. To test these ideas, we developed a comprehensive circadian clock model that extends a recently published model^16^ by incorporating *PER3* and its associated proteins and complexes^15–18, 20, 22, 54, 55^. The genes included in our current model are *PER1, PER2, PER3, CRY1, CRY2, BMAL1, CLOCK*/*NPAS2* (homologs, treated as one gene), and *REV-ERBα,β* (treated as one gene)^13, 14, 56–58^. Figure S1 depicts all modeled human circadian clock network interactions. Together, these genes, their translated protein counterparts, and the kinases CKIδ/ε and GSK3β comprise 208 ordinary differential equations and 100 reaction rates (parameters). Each equation in our model describes the rate of change of an mRNA, protein, or protein complex. Further details of the modeling process are described in the Methods section, and all reactions and rates of the model are provided in Tables S1–S4.

**Figure 3:**
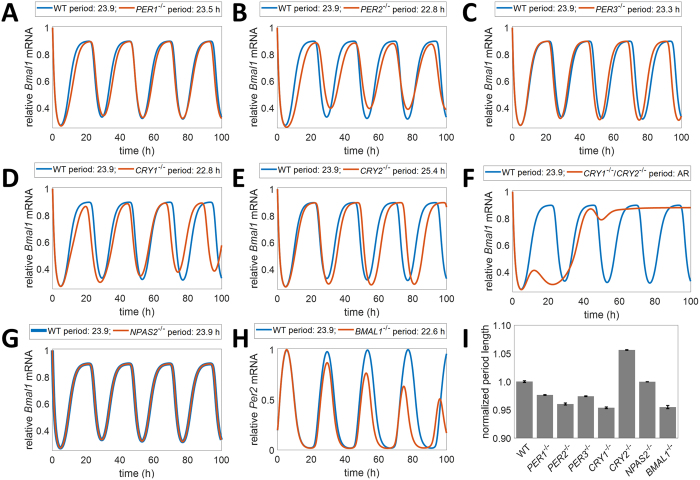
Validation of model fitness using knockout conditions. Concentration data for (**A**–**H**) obtained from a representative example parameter set (See Table S2 for set). (**A**) After *PER1* knockout, *BMAL1* mRNA has a shorter period, as observed experimentally (*p* < 0.001)^55^. (**B**) After *PER2* knockout, *BMAL1* mRNA has a shorter period relative to WT conditions (*p* < 0.001)^55^. (**C**) After *PER3* knockout, *BMAL1* mRNA has a shorter period (*p* < 0.001)^61^. (**D**) After *CRY1* knockout, *BMAL1* mRNA levels display a shorter period (*p* < 0.001)^61^. (**E**) After *CRY2* knockout, *BMAL1* mRNA displays an increase in period length relative to WT conditions (*p* < 0.001)^61^. (**F**) After a double knockout of *CRY1* and *CRY2*, *BMAL1* mRNA levels are arrhythmic ^61^. (**G**) After *NPAS2* knockout, *BMAL1* mRNA levels are unaffected ^61^. Due to the minimal effect of this knockout, the concentration plots overlap. (H) After BMAL1 knockout, PER2 mRNA levels decrease and become arrhythmic. (I) Bar graph of normalized period levels after knockouts. Error bars represent two standard errors from the mean.

**Figure 4:**
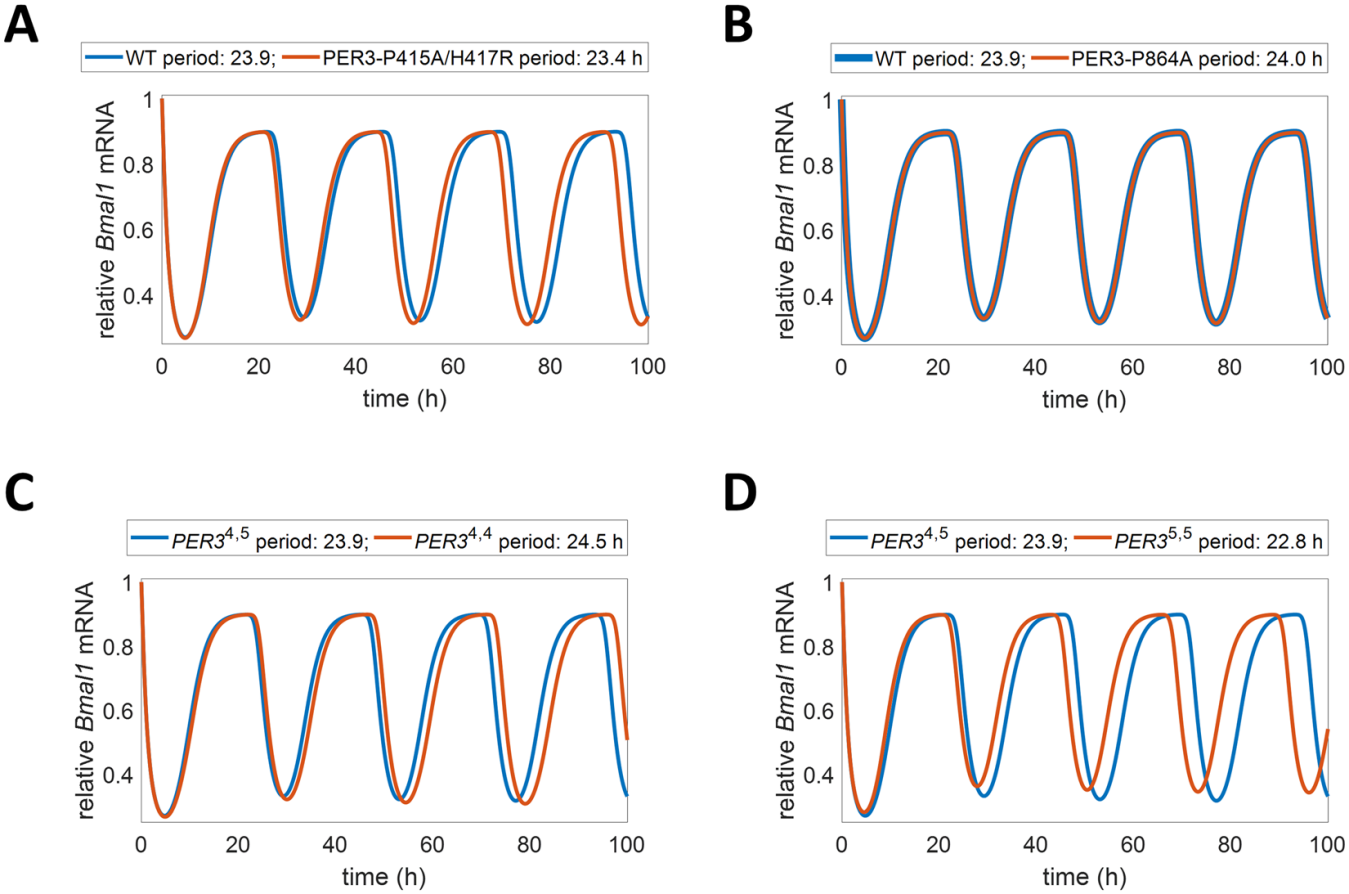
Time series data reflecting SNP/VNTR conditions for a representative individual. (**A**) The model accurately reflects predicted phenotypes for individuals with PER3-P415A/H417R mutations, displaying a shorter period for nuclear *BMAL1* mRNA after an 80% reduction in PER1-PER3 binding rate and an 8-fold increase in PER3 degradation rate (*p* < *0.001*)^30^. (See Tables S2, S5, and S6 for more information about variants). (**B**) The model accurately reflects predicted phenotypes for individuals with PER3-P864A missense mutations, displaying a lengthened period for a representative individual after an 80% decrease in PER3-CKI binding (*p* < *0.001*)^11^. (**C**) The model accurately represents individuals representative of homozygotes of *PER3* ^4,4^ after a 40% decrease in PER3 phosphorylation rate^25^. The *PER3* ^4,4^ homozygous individual displays a lengthened *BMAL1* mRNA period relative to a heterozygous individual (*p* < *0.001*). (**D**) The model accurately represents individuals representative of homozygotes of *PER3* ^5,5^ ^25^. The *PER3* ^5,5^ homozygous individual displays a shortened *BMAL1* mRNA period relative to a heterozygous individual after a 4-fold increase in PER3 phosphorylation rate (*p* < *0.001*)^25^.

Many of the molecular reaction rates used in our model are not experimentally available for this biological system. Therefore, biologically realistic parameter sets for the model were obtained using the Stochastic Ranking Evolutionary Strategy (SRES) parameter estimation algorithm^59^. To find each set, the model simulations were compared to experimental observations from 16 different phenotypes in 12 genetic backgrounds^11, 16, 25, 30, 55, 60, 61^. Knockout conditions were chosen based on their effects on the behavior of multiple mouse strains as well as on gene expression levels of individual human cells (Fig. 3 and Table S1)^55, 60, 61^. Our model successfully predicted the circadian phenotypes (e.g., changes in period length) in all 12 genetic backgrounds, including the dual mutations previously reported in Zhang *et al*. (2016), indicating that the model structure could accurately represent experimental data (Figs 3 and 4, Figure S3, Tables S1 and S7)^30^. In order to test the generality of the model, we used Leave-One-Out analysis^62^. The results of our analysis suggest that our model could accurately predict new *PER3*-related mutations 72 percent of the time, although with further range refinement, this success rate could be increased to over 90 percent (Figure S2). We then used our model and new experimental data to explore the *PER3* mutations studied and their potential links to circadian period length and human mood.

Our comprehensive clock model, the first to incorporate *PER3*, outperformed existing circadian models in its ability to predict all relevant knockout conditions (Table S1), and we anticipate that our model will facilitate hypothesis testing in chronobiology. More importantly, this model allows for an evaluation of the relative impact of alternative mechanistic explanations for the relationships observed in the *PER3* SNP and VNTR studied^11, 25^. Our results suggest that out of the five potential mechanisms studied, PER3-CKIδ/ε and PER3-CRY binding are the most important for period length changes in *PER3*-related diurnal preference. When given significant leeway in parameter ranges, the PER3-CKIδ/ε binding rate was an average of 57 times larger than PER1- or PER2-CKIδ/ε binding rates (*p* < 0.001), while the PER3-CRY binding rate was, on average, 165 times larger than PER1- or PER2-CRY binding rates (*p* < 0.001). In fact, PER3-CKIδ/ε and -CRY binding rates were the only rates that distinguished PER3 from the other PERs; all other rates were approximately the same (Table S2). Thus, our model indicates that the potential mechanism by which *PER3* SNP variants influence anxiety occurs via the interactions of PER3 with CRY and CKIδ/ε proteins.

Although our model does not allow us to test predictions about downstream molecular pathways regulated by the molecular clock, it is likely that the frequency of PER3-CRY or -CKIδ/ε binding affects downstream physiological changes dependent and/or independent of the central circadian clock. One possible candidate would be the dopamine pathway; *CKIδ* is known to regulate dopamine signaling and has been linked to mood disorders in a number of studies^63–65^. Anxiety is associated with an imbalance in dopamine levels^66, 67^, suggesting that altered levels of CKIδ/ε could influence the dopaminergic control of anxiety-related behaviors. *CKIε* is also a member of the canonical Wnt signaling pathway and experimental modifications of this pathway have shown therapeutic benefit in the treatment of mood disorders^68^. Additionally, the CRY proteins are involved in hypothalamus-pituitary-adrenal (HPA) axis regulation^69^, acting as repressors of glucorticoid receptor activity. Glucocorticoids are multifunctional steroid hormones associated with arousal; thus, CRY dysfunction could be linked with the onset of mood disorders via this pathway^70^. In previous studies, *CRY1* and *CRY2* have both been linked with several mood disorders, including bipolar disorder, seasonal affective disorder, and depression^37, 40, 71, 72^. Finally, microarray experiments link the expression of both *CKIε* and *CRY2* to bipolar disorder; valproate, a mood stabilizer, decreased *CKIε* and *CRY2* expression in the amygdala and cotreatment with the stimulant, methamphetamine, prevented this change, suggesting that pathways involving these genes may influence manic-depressive states and related mood disorders^73^.

In summary, our results confirm that polymorphisms in *PER3*, a nonessential clock gene involved in regulating circadian period length, are linked to anxiety, supporting the previously suggested role of *PER3* in regulating human mood^30, 33–40^. The inclusion of *PER3* interactions into our comprehensive circadian clock model enables us to predict circadian phenotypes (e.g., altered period length) associated with mood disorders and to identify critical effects of *PER3* mutations on CKIδ/ε and CRY binding. Thus, our mathematical model provides a molecular framework to explain one potential mechanism underlying the compelling links between *PER3*, circadian rhythms and mood. A better understanding of *PER3* function may help explain how altered sleep/wake rhythms affect anxiety and depression in humans and provide new insights into the development of treatment for affective disorders.

## Methods

### Experimental Data Collection

Students from the Colgate University undergraduate program and from the Johnson School of Management graduate program participated in the study (n = 380; 136 males, 244 females, age range 18–38). An automated survey, including the Horne-Östberg Morningness-Eveningness Questionnaire (MEQ), was administered to each participant. All methods were developed in agreement with the Declaration of Helsinki; procedures and consent forms were approved by the Institutional Review Board at Colgate and Cornell Universities (#FR-F13-07; #1504005518 respectively). Written informed consent was obtained from all participants. MEQ scores, which range from 20 (extreme morning preference) to 80 (extreme evening preference) were age-adjusted using the following formula [78]:

$$\mathrm{MEQ}\,\,{\rm{score}}+0.3512\ast (39.212-{\rm{age}})$$

Participants were also administered the State-Trait Anxiety Inventory (STAI), a commonly used measure of trait and state anxiety^43^. The Trait Anxiety Scale (T-Anxiety) evaluates relatively stable aspects of “anxiety proneness,” including general states of calmness, confidence, and security. We averaged the STAI scores across genotype to test for an effect of genotype on anxiety. Finally, hair samples were collected from each participant to determine participants’ genotypes. Odds ratios were used to test differences in allele frequencies, chi-squared tests were used to test differences in genotype frequecies between chronotypes, t-tests were used to test for differences STAI scores among extreme SNP genotypes, and one-way analyses of variance were used to test differences in MEQ and STAI scores among VNTR and SNP genotypes. A full description of hair sample processing and statistical analysis can be found in Supplementary Information.

### Mathematical Model Structure

We have developed a deterministic differential equation model, in which each equation represents the rate of change of an mRNA, protein, or protein complex. The genes included in our model are *PER1, PER2, PER3, CRY1, CRY2, BMAL1, CLOCK*/*NPAS2*, and *REV-ERBα/β* ^56^. As has been done in previous studies^16, 19^, we treat the gene pairs *CLOCK* and *NPAS2* as well as *REV-ERBα* and *REV-ERBβ* as one gene each based on their similar structure and function^56, 74^. Together, all modeled genes, their translated protein counterparts, and the kinases CKIδ/ε and GSK3β comprise 208 ordinary differential equations and 100 parameters.

Our model is based on the work of Kim & Forger (2012), which originally comprised 70 parameters and 180 differential equations^16, 17^. Our new model includes key circadian clock network changes to improve the previous model:

1. The new model includes all *PER3*-related species, while making the following assumptions:
  - PER3 is phosphorylated by CKIδ/ε^75^.
  - PER3 can only be phosphorylated when in complex with PER1 or PER2^14^.
  - PER2-PER3 complex concentrations are negligible^14^.
  - PER3 interacts with CRY1, CRY2, CLOCK/NPAS2, and BMAL1^14^.
2. The new model also incorporates recent evidence, which suggests that PER1 and PER2 prevent CRY-mediated inhibition of CLOCK phosphorylation^44^.

Due to the complex structure of the model, we use the following naming convention, as has been done in a previous study^16^: all proteins and protein complexes are described in the form x[P][C][K][L][B], where P represents the presence of a PER protein, C represents the presence of a CRY protein, K represents the presence of a bound kinase, L represents location (0 indicates cytoplasm, 1 indicates nucleus), and B represents the presence of a BMAL-CLOCK/NPAS2 complex. For instance, the compound × 10000 represents an unphosphorylated PER1 protein located in the cytoplasm of the cell. A representative equation is described below:

$$\begin{array}{rcl}x10000 & = & tlpo\ast McPo-upuo\ast x10000-ac\ast x00100\ast x10000\\  &  & +dc\ast x10100-an\ast x70000\ast x10000+dn\ast x80000\end{array}$$

In the above equation, *tlpo* indicates translation rate of cytoplasmic *PER1* mRNA (*McPo*). Once PER1 protein has been generated, it then may degrade (*upuo*), may bind to CKIδ/ε (*ac*), or may unbind from the PER1-CKI δ/ε complex (*dc*). It also may bind to PER3 (*an*), or may unbind from the PER1-PER3 unphosphorylated complex (*dn*). All parameters and equations are described in full in Tables S2, S3 and S4.

### Numerical Simulation

Our deterministic model is solved numerically using Euler’s method. Euler’s method increments the time in the chosen step size (0.005 hours), and updates mRNA, protein, and protein complex levels at each iteration using the rates of change provided by the model (Table S2). To simulate a knockout mutation, we set the transcription rate of the corresponding clock gene to zero (Table S5). To simulate a SNP or VNTR condition, we parameterized the appropriate biochemical rate, as described in Table S6.

Euler’s method was chosen due to its speed. We confirmed the accuracy of this method in two ways. First, we varied the step-size, comparing our chosen step-size of 0.005 hours to a step-size of 0.0025 hours. The average relative error between the two simulations was 0.054%, and no individual simulation differed by more than 0.34%. The average difference in period calculated from the two simulations was 0.05%, and no individual period differed by more than 0.14%. These small variations did not change our main findings. Second, we compared the Euler method to ode15s, a MATLAB-based variable step-size numerical solver for stiff systems. Ode15s is known to be an accurate numerical solver, and this method has been used in a previous study of large, nonlinear circadian clock modeling^16^. We compared the two solvers using a step size of 0.005 for the Euler method. The average relative error between the two simulations was 0.19%, and no individual simulation differed by more than 0.37%. The average period difference was 0.091%, and no individual period differed by more than 0.23%. Again, these small variations did not change our main findings.

### Parameter Estimation

Most of the reaction rates in the human circadian clock network have not been measured experimentally due to technical limitations. We employed a parameter search algorithm to identify parameter values that could reproduce experimental observations. Experimental knockout conditions were obtained from studies performed in transgenic mice and in isolated human cells; all mouse conditions were chosen based on their resemblance to gene expression patterns in human cell lines (Table S1)^55, 60, 61^. All SNP and VNTR data were obtained from peer-reviewed sources linking human *PER3* to specific variations in sleeping patterns^11, 25, 30^. For a full list of experimental conditions used, see Tables S5 and S6.

Parameter search was performed using the stochastic ranking evolutionary strategy (SRES) algorithm^59^, which looked for suitable parameter sets able to fit experimental conditions described in Tables S5 and S6. Previous research indicates that SRES performs better than other global parameter estimation algorithms, such as simulated annealing, in large-scale biological systems^62, 76^. Parameters retained from Kim and Forger (2012) were allowed up to 50 percent variability from previous ranges^16^, while new parameters were given freedom within biologically realistic limits. To produce the biologically feasible parameter ranges described in Table S2, parameter ranges were further refined based on the results of initial parameter searches (See Supplementary Information).

### Oscillation Features and Model Conditions

The experimental observations used in this study were related to the amplitude and period of circadian gene transcript oscillations^11, 25, 30, 55, 60, 61^. To calculate the amplitude of oscillations in our model, we used the difference between the last peak and trough of the nuclear *BMAL1* mRNA oscillations (except in the case of *BMAL1* knockdown, in which *PER2* nuclear mRNA oscillations were used). Period was calculated as the time difference between the last two peaks. Both *BMAL1* and *PER2* are circadian clock genes known to be both essential and cyclical, and therefore were used as markers of period^14, 77^. The periods calculated from these two genes differed by less than 0.8% (mean ± 2 standard errors: 0.730 ± 0.037%). However, using other necessary clock genes did not appear to significantly change our findings; the maximum difference in period calculated from *BMAL1, PER1, PER2*, and *CRY1* was less than 2% (1.51 ± 0.14%).

All experimental conditions are based on period and amplitude variations in *BMAL1* and *PER2* mRNA oscillations. Extreme morningness or FASPD were each defined as a 2–6% decrease in the average period of nuclear *BMAL1* mRNA. Extreme eveningness or DSPD were each defined as a 2–6% increase in the average period of nuclear *BMAL1* mRNA. Further details of the model conditions are specified in Tables S4, S5 and S6.

### Model Validation

To test the predictive accuracy of the model and the robustness of the estimated parameters, we performed Leave-One-Out Analysis. This model validation technique requires evaluating the effects of removing individual experimental conditions from the parameter estimation process. There were 16 experimental conditions. In each iteration of Leave-One-Out Analysis, one condition was left out for testing, and all the remaining experimental conditions were used for parameter estimation. Predictive accuracy of our model was calculated as the average accuracy of the iterations of Leave-One-Out Analysis on the test data. We performed the Leave-One-Out Analysis in two ways: once without extensive parameter range refinement, and once with significant range refinement to determine whether our results could be improved by precise experimental measurement of biochemical rates. Details of the Leave-One-Out Analysis are provided in Supplementary Information, and results are shown in Figure S2.

To further test the predictive accuracy of the model, we performed a Leave-Sets-Out Analysis. We re-ran parameter estimation without including conditions relating to SNPs or VNTRs (Tables S6 and S7). Using our newly estimated parameters, we tested each SNP and VNTR by varying PER3 phosphorylation, degradation, and CKIδ/ε, PER1, and CRY binding rates to determine these mutations’ individual effects on *BMAL1* nuclear mRNA period. Results are shown in Figure S3.

### Coding

The codes for the study have been implemented in C++, Python, and MATLAB. C++ was used because of its speed and Python and MATLAB because of their user-friendliness and superior data processing features. Each simulation code written in C++ took less than one minute (performed on a single processor of a computer cluster with 19 nodes, 248 processors, and 24 gigabytes of RAM per node). Each SRES parameter estimation run with 5,000 generations, 3 parents, and a population size of 20 took approximately five hours (using one node with eight processors and code written in C++).

### Code Availability

The code and read-me files are available upon request.

### Data Availability

All data will be available in the DRYAD repository following acceptance for publication.

## Electronic supplementary material

Supplementary Information
