# Supplementary material for: Circadian Clock Model Supports Molecular Link Between PER3 and Human Anxiety
Source: Sci Rep. 2017 Aug 31;7:9893. doi: 10.1038/s41598-017-07957-4 (PMC5579000; doi:10.1038/s41598-017-07957-4)
Supplement: Supplementary file 1 — Supplementary Information [file 41598_2017_7957_MOESM1_ESM.pdf]

Supplementary Information

Supplementary Methods.

MEQ Chronotype Analysis. Students from the Colgate University undergraduate program and from the Johnson School of Management graduate program participated in the study (n=380; 136 males, 244 females, age range 18-38). The demography of the population is approximately: 69% Caucasian, 10% Latino, 9% Asian, 5% African American, 56% female, and mixed socio-economic status. Our participants were sampled across this ethnic and socio-economic diversity. Our sampling was not discipline-specific. An automated survey, including the Horne-Östberg Morningness-Eveningness Questionnaire (MEQ), was administered to each participant. The MEQ consists of 19 questions that assess diurnal preference (i.e. timing of daytime activities, sleeping habits, hours of peak performance, times of maximum alertness, etc.). Individuals with high scores represent moderate (>59) or extreme (>70) morningness chronotypes while individuals with low scores represent moderate (<41) or extreme (<30) eveningness chronotypes. The correlation between MEQ scores and sleep-wake cycles has been validated in numerous previous studies.[1,2] All methods were developed in agreement with the Declaration of Helsinki; procedures and consent forms were approved by the Institutional Review Board at Colgate and Cornell Universities (#FR-F13-07; #1504005518). All methods were carried out in accordance with the approved guidelines. Informed consent was obtained from all individuals before samples were taken. MEQ scores were age-adjusted using the following formula: $\text{MEQ score} + 0.3512 * (39.212 - \text{age})$.[3]

STAI (State Trait Anxiety Index). The State-Trait Anxiety Inventory (STAI) is a commonly used measure of trait and state anxiety.[4] We used STAI to measure trait anxiety. The Trait Anxiety Scale (T-Anxiety) evaluates relatively stable aspects of "anxiety proneness," including general states of calmness, confidence, and security. We averaged the STAI scores across genotype to test for an effect of genotype on sleep quality.

Genotyping. Hair samples were collected from each participant. Following digestion of hair at 37°C for 24 hours, DNA was extracted and purified with the Qiagen DNAeasy Micro Kit. Genotyping for PER3 SNP rs228697 was performed using a TaqMan SNP Genotyping assay (Applied Biosystems, Foster City, CA) on an ABI 3700HT real-time qPCR instrument. To measure the VNTR length polymorphism of 54 base pairs (bp) in exon 18 of the PER3 gene, we used a fragment length analysis on an ABI 3100 sequencer. The following PCR primers were used with the forward primer fluorescently labeled with 6-FAM : forward, 5'-CAAAATTTTATGACACTACCAGAATGGCTGAC-3', and reverse, 5'-AA CCTTGTACTTCCACATCAGTGCCTGG-3'.[5] The PCR was performed in a 25-uL volume using Qiagen PCR Mastermix. A positive DNA control and a negative water control were included with each PCR plate of samples. The PCR cycling conditions were 3 min. at 94°C, followed by 35 cycles of 45 sec. at 94°C, 45 sec. at 58°C, and 45 sec. at 72°C, with a final step at 72°C for 3 min. PER3 alleles were separated by capillary electrophesis and sized using ABI ROX standards.[6]

Statistical Analysis. Hardy-Weinberg Equilibrium (HWE) was estimated using Genepop. Chi-squared tests were performed to compare the genotype and allele frequencies for both markers among the chronotype groups (morning, intermediate, and evening types). The sex-adjusted ORs and 95% CIs with Bonferroni correction were calculated to evaluate the rs228697 SNP and the *PER3* VNTR frequency across the three diurnal preference groups. One-way analysis of variance was performed to compare age-adjusted MEQ scores between subjects with genotype CC or CG and those with genotype GG for rs228697 and between subjects with $PER3^{4,4}$ or $PER3^{4,5}$ genotypes and $PER3^{5,5}$ genotypes. One-way analyses of variance were performed to compare STAI scores between subjects with genotype CG or GG and those with genotype CC for rs228697 and between subjects with $PER3^{4,4}$, $PER3^{4,5}$, and $PER3^{5,5}$ genotypes. A student's t-test assuming unequal variances was used to compare parameter values in the model. All statistical analyses were performed using MATLAB and SPSS.

Evaluating SNP and VNTR data in the Mathematical Model. All SNP and VNTR data was obtained from peer-reviewed sources linking *PER3* to specific variations in sleeping patterns.[7–9] Assumptions regarding molecular effects (Table S6) were made using the PER3 protein structure described in Gustafson & Partch (2014).[10] Because PER3-P415A/H417R has been shown to decrease protein stability and reduce the protein's ability to stabilize PER1 and PER2, we assumed that this mutation would increase PER3 protein degradation rates (upuh, uph) and decrease PER1-PER3 binding (an) (our model assumes negligible PER2-PER3 binding).[9,11] Because 32 percent of amino acid residues in the repeat region studied were potential sites for phosphorylation, we assumed that $PER3^4$ homozygotes and $PER3^5$ homozygotes had decreased and increased phosphorylation, respectively, relative to heterozygotes.[7] Finally, because PER3-P864A occurs within a CKI-binding domain, changing the hydropathy and potentially the secondary structure of the site, we assumed that the mutation could potentially decrease PER3-CKIδ/ε binding.[8]

Leave-one-out analysis. To test the predictive accuracy of the model and the robustness of the estimated parameters, we evaluated the effects of removing individual conditions from parameter estimation. There were 16 conditions, one of which (the wild-type condition, ensuring sustained oscillations and an appropriate period length for our simulations) was never removed. Each of the other 15 conditions was removed one at a time. Two different approaches were used. First, we refined the parameter ranges using all conditions until we saw no significant change in the ranges, as indicated by an average change of less than 0.2 percent for all parameters and a maximum change of less than five percent for any individual parameter. Using these ranges, we removed one condition at a time and estimated the model parameters. Then, these parameter sets were used to check whether the missing condition was satisfied. This procedure was repeated twice more, and the average of the three trials is reported in Figure S2.

To test whether our results could be improved by further parameter refinement or by accurate and precise experimental measurement of the biochemical rates, we repeated the leave-one-out analysis with more restricted parameter ranges. In this approach, we chose new parameter ranges centered around the parameter sets that passed all 16 conditions as follows:

> Let Π be the ith parameter from any parameter set that passed all conditions, and let L_i and U_i be the lower and upper bounds for the ith parameter in the ranges used in the first approach. Then the new lower bound is defined as $\Pi - 0.025 * (U_i - L_i)$ and the new upper bound is defined as $\Pi + 0.025 * (U_i - L_i)$. In other words, no parameter value was allowed to vary by more than five percent from the provided values.

We next performed the leave-one-out analysis for three randomly selected parameter ranges. Each time, we used three randomly chosen sets of 500 parameter sets estimated from within the parameter ranges described above (Figure S2).

Leave-Sets-Out Analysis. We next tested the effects of varying PER3 degradation, binding, and phosphorylation rates on model period. First, we found 1,000 parameter sets that fit only the wildtype and seven experimental knockout conditions described in Table S5. The SNP and VNTR conditions described in Table S6 were not used in the initial parameter estimation. Instead, each of the parameters relating to SNP or VNTR conditions (an, acph, and hoho, as seen in Table S2) were then decreased by 20, 40, 60, 80, or 100 percent or increased (upuh, uph, upoh, upuoh, hoho) by 4, 8, 12, 16, or 20 times to simulate each of the SNP/VNTR conditions (Table S6). This procedure was repeated three times.

Supplementary Figures.

Figure S1. Schematic of the human circadian clock model. Only relevant species are shown. The model consists of a core negative feedback loop and an additional negative feedback loop. The core loop is made up of the activator complex BMAL1-CLOCK, which activates transcription of *PER*, *REV-ERB*, and *CRY* species. The PER and CRY proteins and complexes then feed back to inhibit BMAL1-CLOCK, thus inhibiting their own transcription. The smaller, secondary negative feedback loop consists of the *REV-ERB* genes, whose transcription *BMAL1* promotes. The REV-ERB proteins then inhibit *BMAL1* transcription, completing the feedback loop.

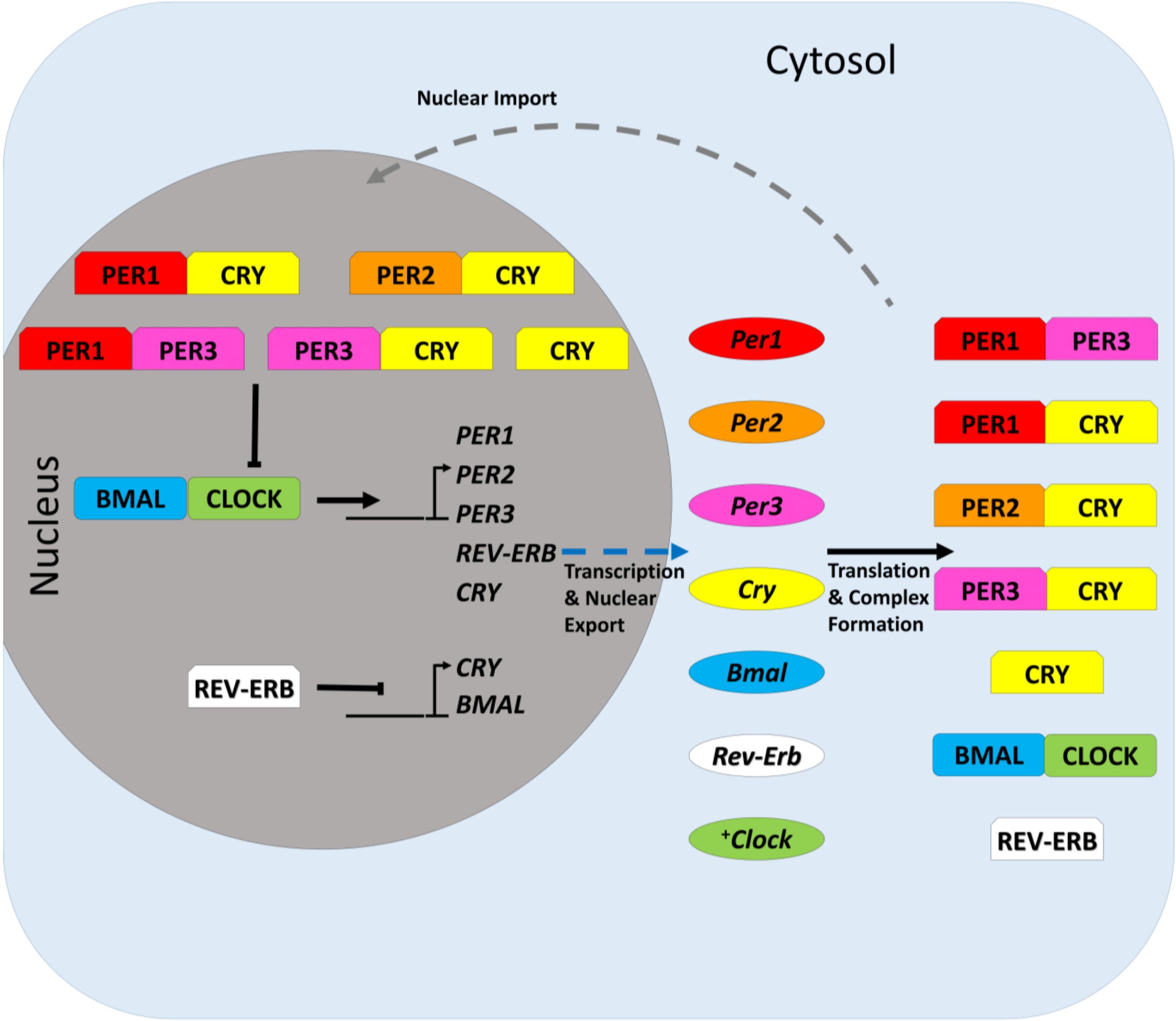

Figure S2. Leave-one-out analysis for mutant conditions. Error bars represent 2 standard errors from the mean. (A) Prior to range refinement, leave-one-out results for knockout conditions varied widely, with an average success rate of 58.5% for each condition. (B) Prior to range refinement, leave-one-out results for SNP and VNTR conditions varied widely, with an average success rate of 72.3% for each condition. (C) After further refinement of the parameter ranges, leave-one-out success rate of knockout conditions increased to an average of 94.8%. (D) After further refinement of the parameter ranges, leave-one-out success rate of SNP and VNTR conditions increased to an average of 98.6%, supporting the idea that additional experimental data could increase the predictive power of this model.

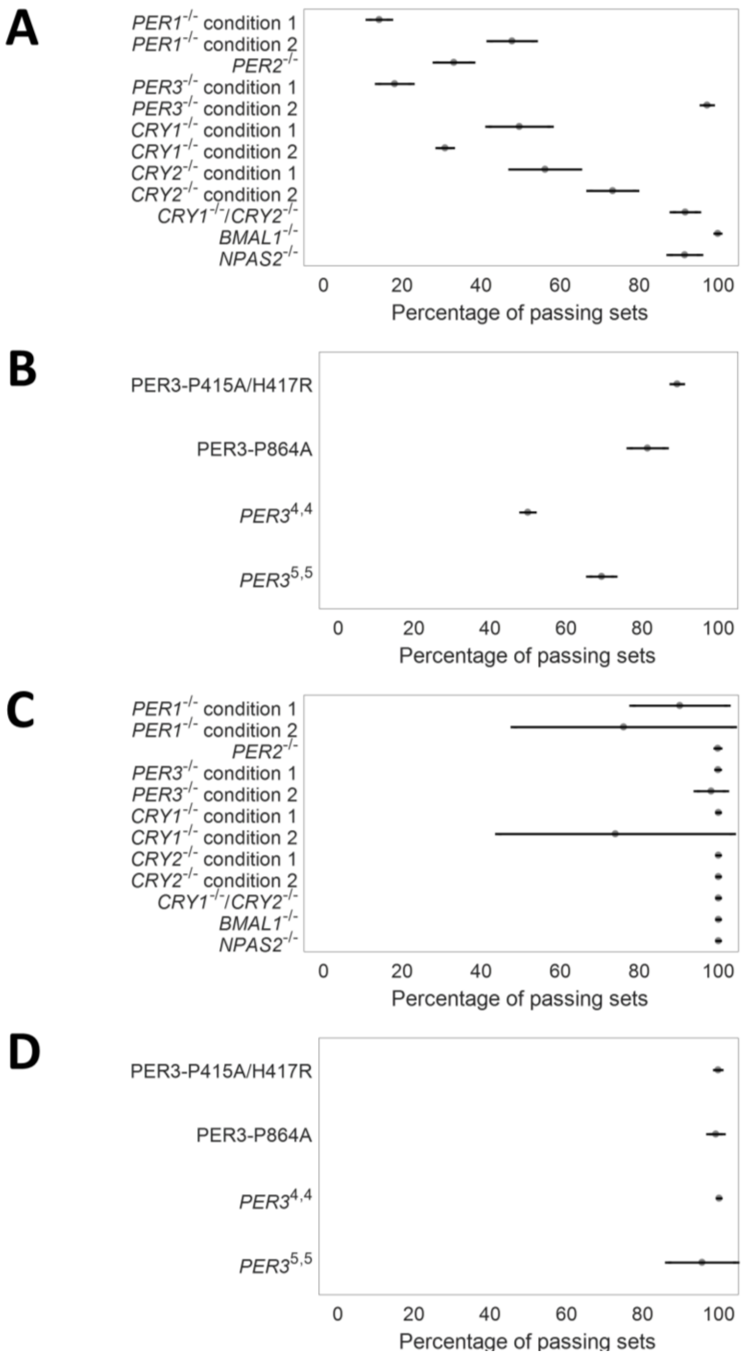

Figure S3. Testing model fitness to SNP/VNTR conditions. Error bars represent two standard errors from the mean. (A) The model accurately represents homozygotes of *PER3*^{4,4} and *PER3*^{5,5}. After decreasing PER3 phosphorylation by 20, 40, 60, and 80 percent, *PER3*^{4,4} homozygous individuals exhibited lengthened periods for *Bmal1* mRNA, representative of morning preference ($p < 0.002$). (B) After increasing PER3 phosphorylation by 4, 8, 12, and 16 times, *PER3*^{5,5} homozygous individuals exhibited shorter periods for *Bmal1* mRNA, representative of extreme evening preference or of a DSP phenotype ($p < 0.04$). (C) The model accurately reflects predicted phenotypes for individuals with PER3-P415A/H417R mutations, which may lead to decreased PER1/PER3 binding and/or increased PER3 degradation rate.[9] (See Table S6 for more information about variants). After decreasing PER1-PER3-binding by 20, 40, 60, and 80 percent, representative individuals have shortened periods for *Bmal1* mRNA levels, representative of the FASP phenotype ($p < 0.004$). (D) After increasing PER3 degradation rates by 4, 8, 12, 16, and 20 times, representative individuals have shortened periods for *Bmal1* mRNA levels, representative of the FASP phenotype ($p < 0.008$). (E) The model accurately reflects predicted phenotypes for individuals with PER3-P864A mutations.[8] After decreasing Casein Kinase 1 (CKI) binding in PER3 species by 20, 40, 60, and 80 percent, representative individuals exhibited lengthened periods for *Bmal1* mRNA, representative of eveningness ($p = 0.13$).

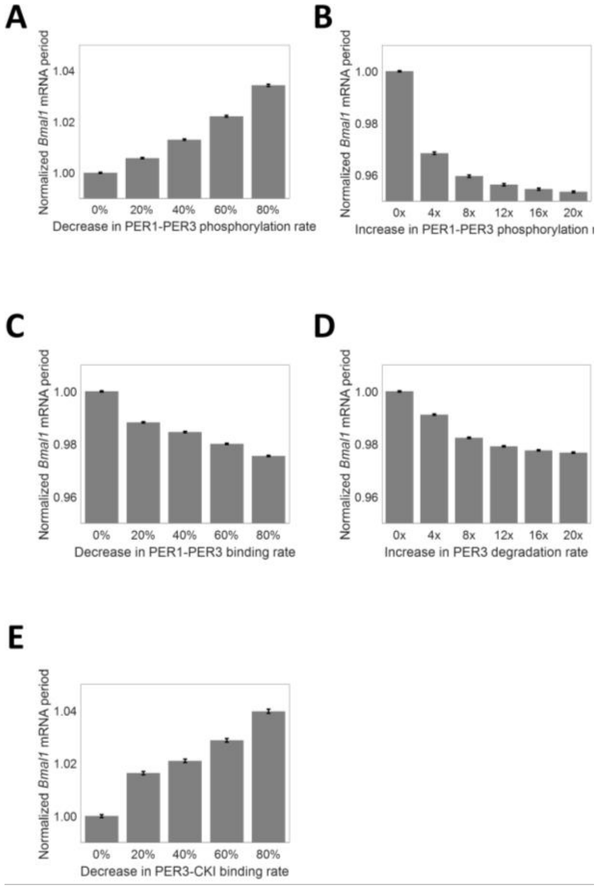

Supplementary Tables

Table S1. Comparison of new model predictions with previous model predictions on the phenotypes of circadian gene knockouts. "Short" represents shorter average period relative to wild-type, "long" represents longer average period relative to wild-type, "AR" represents arrhythmicity, "WT" represents a wild-type phenotype, and "N/A" indicates the model did not analyze this model condition. Red text indicates an incorrect prediction of effects observed in the biological system. Our model outperforms existing models in predicting the knockout conditions.

**Gene**	**Predicted effects on *Bmal1* mRNA**	**Mouse strains exhibiting behavioral effect**	**Cells exhibiting gene expression level effects**	**New Model**	**Jolley *et al* (2014)**[12]	**Kim & Forger (2012)**[13]	**Ogawa *et al* (2011)**[14]	**Relógio *et al* (2011)**[15]	**Mirsky *et al* (2009)**[16]
PER1$^{-/-}$	short or AR	*Per1*^{brdm1}, *Per1*^{luc}, *Per1*$^{-/-}$ [17]	**Human:** MMH-D3 hepatocytes, U20S Osteosarcoma cells[18]<br><br>***Per2***^{Luc} **Mice:** individual SCN neurons, lung explants, fibroblast tissue, individual fibroblasts[19]	**short**	N/A	**WT**	**short**	**AR**	**AR**
PER2$^{-/-}$	short	*Per2*^{brdm}, *Per2*^{ldc};[17] *Per2*^{Luc} [19]	**Human**: 3T3 fibroblasts, MMH-D3 hepatocytes[18]	**short**	N/A	**AR**	**short**	**AR**	**AR**
PER3$^{-/-}$	short	*Per3*$^{-/-}$ [17]	**Human:** 3T3 fibroblasts, 3T3-L1 adipocytes, MMH-D3 hepatocytes, U20S osteosarcoma cells[18]<br><br>***Per2***^{Luc} **Mice:** lung explants and fibroblasts[19]	**short**	N/A	N/A	N/A	N/A	N/A
CRY1$^{-/-}$	short	*Cry1*$^{-/-}$;[17] *Per2*^{Luc} [19]	**Human:** U20S osteosarcoma cells[18]<br><br>***Per2***^{Luc} **Mice:** SCN explants[19]	**short**	**AR**	**short**	**short**	**long**	**AR**
CRY2$^{-/-}$	long	*Cry2*$^{-/-}$;[17] *Per2*^{Luc} [19]	**Human:** 3T3 fibroblasts, 3T3-L1 adipocytes, MMH-D3 hepatocytes, U20S osteosarcoma cells[18]<br><br>***Per2***^{Luc} **Mice:** SCN explants, individual SCN neurons, lung explants, fibroblast tissue, individual fibroblasts[19]	**long**	N/A	**long**	**short**	**long**	**long**
CRY1/CRY2$^{-/-}$	AR	*Cry1*$^{-/-}$/*Cry2*$^{-/-}$ [17]		**AR**	**AR**	N/A	N/A	**AR**	**AR**
NPAS2$^{-/-}$	WT		**Human:** 3T3 fibroblasts, 3T3-L1 adipocytes, MMH-D3 hepatocytes[18]	**WT**	N/A	**WT**	N/A	N/A	N/A

Table S2. Description of model parameters and ranges.

Symbol	Parameter Name	Pre-Parameter-Search Range (Lower bound, upper bound)	Post-Parameter-Search Range (Lower bound, upper bound)	Further Range Refinement for Leave-One-Out Analysis (Lower bound, upper bound)	Example Parameter Set	Average ± 2 Standard Errors
**1. trPo**	Transcription rate constant for *PER1*	12.960, 38.880	22.475, 30.045	26.688, 27.508	23.111	25.670 ± 0.407
**2. trPt**	Transcription rate constant for *PER2*	22.427, 67.281	34.463, 57.793	38.421, 40.041	44.456	39.947 ± 0.817
3. trPh	Transcription rate constant for *PER3*	0.000, 60.000	30.242, 56.164	42.123, 44.283	43.203	46.150 ± 0.889
**4. trRo**	Transcription rate constant for *CRY1*	11.537, 34.612	15.601, 23.235	21.494, 22.078	22.287	20.707 ± 0.393
**5. trRt**	Transcription rate constant for *CRY2*	19.970, 59.911	23.235, 35.268	25.056, 25.791	27.129	27.910 ± 0.513
**6. trB**	Transcription rate constant for *BMAL1*	23.052, 69.156	24.538, 44.673	38.260, 40.125	34.364	38.640 ± 0.807
**7. trNp**	Transcription rate constant for *NPAS2*	0.165, 0.495	0.173, 0.247	0.199, 0.214	0.190	0.195 ± 0.003
**8. trRev**	Transcription rate constant for *REV-ERBs*	51.462, 154.385	70.552, 129.262	105.649, 109.357	99.432	106.460 ± 1.692
**9. tlpo**	Translation rate constant for PER1	0.905, 2.715	1.430, 2.265	1.605, 1.655	2.043	2.116 ± 0.023
10. tlpt	Translation rate constant for PER2	0.000, 6.000	3.613, 5.757	3.669, 3.808	5.162	4.502 ± 0.102
11. tlph	Translation rate constant for PER3	0.000, 6.000	3.650, 5.894	5.468, 5.585	5.214	4.849 ± 0.096
**12. tlro**	Translation rate constant for CRY1	2.519, 7.558	3.630, 6.219	5.026, 5.205	5.185	5.106 ± 0.123
13. tlrt	Translation rate constant for CRY2	0.000, 6.000	3.212, 5.434	4.209, 4.357	4.588	4.788 ± 0.082
**14. tlb**	Translation rate constant for BMAL	0.265, 0.796	0.320, 0.581	0.566, 0.581	0.457	0.476 ± 0.009
**15. tlc**	Translation rate constant for CLOCK	2.323, 6.969	3.556, 6.280	6.092, 6.278	5.080	4.845 ± 0.159
**16. tlnp**	Translation rate constant for NPAS2	0.625, 1.876	0.760, 1.346	0.844, 0.899	1.035	1.085 ± 0.029
**17. tlrev**	Translation rate constant for REV-ERBs	4.454, 13.361	8.154, 12.426	9.686, 9.926	11.648	11.304 ± 0.184
**18. agp**	Binding rate constant for PER2 to GSK3β	0.698, 2.094	1.236, 1.822	1.722, 1.810	1.766	1.506 ± 0.039
**19. ag**	Binding rate constant for REV-ERBs to GSK3β	0.081, 0.244	0.081, 0.135	0.101, 0.105	0.104	0.105 ± 0.002
**20. dg**	Unbinding rate constant for REV-ERBs to GSK3β	1.468, 4.403	2.286, 3.652	2.996, 3.099	3.266	3.046 ± 0.073
**21. ac**	Binding rate constant for PER1,2 to CKIε/δ	0.023, 0.068	0.024, 0.041	0.033, 0.034	0.032	0.031 ± 0.001
22. acph	Binding rate constant for PER3 to CKIε/δ	0.000, 7.000	1.114, 2.070	1.552, 1.632	1.592	1.692 ± 0.037

**23. dc**	Unbinding rate constant for PER to CKIε/δ	0.054, 0.162	0.098, 0.155	0.150, 0.153	0.120	0.129 ± 0.003
**24. ar**	Binding rate constant for PER1,2 to CRY	0.012, 0.035	0.025, 0.033	0.027, 0.027	0.029	0.027 ± 0.000
**25. dr**	Unbinding rate constant for PER1,2 to CRY	0.303, 0.908	0.352, 0.585	0.354, 0.379	0.450	0.436 ± 0.010
26. arph	Binding rate constant for PER3 to CRY	0.000, 7.000	3.163, 5.875	4.406, 4.632	4.519	4.360 ± 0.130
27. drph	Unbinding rate constant for PER3 to CRY	0.000, 7.000	0.428, 0.795	0.597, 0.627	0.612	0.637 ± 0.018
28. an	Binding rate constant for PER1 to PER3	0.000, 7.000	1.503, 2.790	2.093, 2.200	2.146	2.071 ± 0.059
29. dn	Unbinding rate constant for PER1 to PER3	0.000, 7.000	3.724, 6.532	5.187, 5.453	5.320	5.594 ± 0.112
**30. bbin**	Binding rate constant for PER to BMAL/CLOCK-NPAS2	3.463, 10.390	4.933, 7.064	6.872, 7.224	7.048	6.059 ± 0.153
**31. unbbin**	Unbinding rate constant for PER to BMAL/CLOCK-NPAS2	0.065, 0.195	0.134, 0.195	0.187, 0.197	0.192	0.172 ± 0.004
**32. cbbin**	Binding rate constant for CRY to BMAL-CLOCK/NPAS2	3.300, 9.899	4.836, 7.252	5.777, 6.074	5.926	5.922 ± 0.121
**33. uncbbin**	Unbinding rate constant for CRY to BMAL-CLOCK/NPAS2	0.152, 0.456	0.220, 0.336	0.252, 0.265	0.258	0.261 ± 0.005
**34. cbin**	Binding rate constant for BMAL to CLOCK/NPAS2	0.023, 0.068	0.023, 0.042	0.031, 0.033	0.032	0.030 ± 0.010
**35. uncbin**	Unbinding rate constant for BMAL to CLOCK/NPAS2	3.636, 10.908	6.456, 10.845	8.992, 9.453	9.223	8.459 ± 0.254
**36. bin**	Normalized binding rate constant for CLOCK/NPAS2 to *CRY1* E-box	3.486, 10.457	6.792, 10.184	9.461, 9.946	9.703	8.573 ± 0.203
**37. unbin**	Normalized unbinding rate constant for CLOCK/NPAS2 to *CRY1* E-box	0.128, 0.383	0.207, 0.375	0.281, 0.296	0.289	0.282 ± 0.007
38. binpo	Normalized binding rate constant for CLOCK/NPAS2 to *PER1* E-box	0.000, 12.000	5.433, 10.090	7.567, 7.955	7.761	7.356 ± 0.219
39. unbinpo	Normalized unbinding rate constant for CLOCK/NPAS2 to *PER1* E-box	0.000, 12.000	1.548, 2.875	2.156, 2.267	2.212	2.226 ± 0.063

40. binpt	Normalized binding rate constant for CLOCK/NPAS2 to *PER2* E-box	0.000, 12.000	7.259, 11.193	10.196, 10.491	10.371	9.530 ± 0.190
41. unbinpt	Normalized unbinding rate constant for CLOCK/NPAS2 to *PER2* E-box	0.000, 12.000	1.482, 2.752	2.064, 2.170	2.117	2.123 ± 0.060
42. binph	Normalized binding rate constant for CLOCK/NPAS2 to *PER3* D-box	0.000, 12.000	4.087, 7.590	5.692, 5.984	5.838	5.732 ± 0.149
43. unbinph	Normalized unbinding rate constant for CLOCK/NPAS2 to *PER3* D-box	0.000, 12.000	5.087, 7.209	5.407, 5.684	5.546	5.868 ± 0.147
**44. binc**	Normalized binding rate constant for CLOCK/NPAS2 to *CRY2* E-box	0.140, 0.421	0.291, 0.540	0.405, 0.425	0.415	0.417 ± 0.013
**45. unbinc**	Normalized unbinding rate constant for CLOCK/NPAS2 to *CRY2* E-box	0.004, 0.013	0.009, 0.013	0.013, 0.013	0.013	0.012 ± 0.000
**46. binr**	Normalized binding rate constant for CLOCK/NPAS2 to *REV-ERBs* E-box	3.077, 9.232	3.077, 4.251	3.188, 3.352	3.270	3.642 ± 0.074
**47. unbinr**	Normalized unbinding rate constant for CLOCK/NPAS2 to *REV-ERBs* E-box	1.455, 4.365	1.835, 2.650	1.988, 2.090	2.039	2.083 ± 0.031
48. bincryb	Normalized binding rate constant for CRY to unphosphorylated BMAL-CLOCK/NPAS2	0.000, 7.000	1.121, 1.606	1.561, 1.641	1.601	1.470 ± 0.026
49. unbincryb	Normalized unbinding rate constant for CRY to unphosphorylated BMAL-CLOCK/NPAS2	0.000, 7.000	3.289, 4.409	3.307, 3.477	3.392	3.723 ± 0.061
**50. binrevb**	Normalized binding rate constant for REV-ERBs to *BMAL* RORE	0.003, 0.009	0.006, 0.009	0.009, 0.009	0.008	0.008 ± 0.000
**51. unbinrevb**	Normalized unbinding rate constant for REV-ERBs to *BMAL* RORE	2.653, 7.958	3.284, 5.598	4.751, 4.898	4.691	4.448 ± 0.099
**52. binrev**	Normalized binding rate constant for	0.006, 0.018	0.011, 0.016	0.016, 0.016	0.016	0.015 ± 0.000

	REV-ERBS to *CRY1* RORE					
**53. unbinrev**	Normalized unbinding rate constant for REV-ERBS to *CRY1* RORE	5.487, 16.461	8.268, 15.354	11.007, 11.447	11.811	10.923 ± 0.299
54. binrevn	Normalized binding rate constant for REV-ERBS to Npas2 RORE	0.000, 12.000	0.093, 0.173	0.129, 0.136	0.133	0.126 ± 0.004
55. unbinrevn	Normalized unbinding rate constant for REV-ERBS to *NPAS2* RORE	0.000, 12.000	3.197, 5.937	4.453, 4.681	4.567	4.920 ± 0.125
**56. tmc**	Rate constant for folding and nuclear export of *Per1/2*, *Cry1/2*, *Bmal,* and *Npas2* mRNA	0.082, 0.246	0.151, 0.208	0.202, 0.205	0.160	0.164 ± 0.003
**57. tmcrev**	Rate constant for folding and nuclear export of *Rev-erb*s mRNA	4.632, 13.895	8.036, 13.533	11.932, 12.359	11.480	11.639 ± 0.227
**58. nl**	Nuclear localization rate constant for proteins bound to PER	0.322, 0.965	0.676, 0.965	0.919, 0.934	0.880	0.810 ± 0.016
**59. ne**	Nuclear export rate constant for proteins bound to PER	0.013, 0.040	0.015, 0.027	0.024, 0.025	0.020	0.021 ± 0.001
**60. nlrev**	Nuclear localization rate constants for proteins bound to REV-ERBs	4.819, 14.456	6.327, 9.817	7.363, 7.741	7.552	7.554 ± 0.175
**61. nerev**	Nuclear export rate constant for proteins bound to REV-ERBs	0.008, 0.023	0.011, 0.020	0.016, 0.017	0.015	0.015 ± 0.000
**62. nlbc**	Nuclear localization rate for BMAL-CLOCK/NPAS2	2.633, 7.898	3.035, 5.637	4.228, 4.445	4.336	4.497 ± 0.127
**63. lne**	Nuclear export rate constant for unbound kinases	0.297, 0.892	0.449, 0.707	0.657, 0.677	0.544	0.598 ± 0.012
**64. hoo**	CKIε/δ phosphorylation rate for PER1	0.264, 0.791	0.354, 0.597	0.398, 0.410	0.506	0.489 ± 0.011
**65. hto**	CKIε/δ phosphorylation rate for PER2	1.228, 3.684	2.047, 3.667	2.069, 2.175	1.584	3.024 ± 0.072
66. hoho	CKIε/δ phosphorylation rate for PER1-PER3 complex	0.000, 3.000	0.340, 0.489	0.367, 0.386	0.376	0.410 ± 0.008
**67. phos**	Phosphorylation rate constant for BMAL-CLOCK/NPAS2	0.146, 0.437	0.197, 0.311	0.232, 0.244	0.239	0.238 ± 0.004

**68. trgto**	Increase rate of GSK3β activity	0.322, 0.967	0.590, 0.950	0.821, 0.863	0.842	0.799 ± 0.019
**69. ugto**	Decrease rate of GSK3β activity	0.031, 0.094	0.047, 0.068	0.066, 0.069	0.068	0.061 ± 0.001
**70. umPo**	Degradation rate constant for *PER1*	0.383, 1.150	0.383, 0.460	0.439, 0.443	0.427	0.410 ± 0.003
**71. umPt**	Degradation rate constant for *PER2*	0.294, 0.883	0.345, 0.437	0.378, 0.383	0.405	0.418 ± 0.003
72. umPh	Degradation rate constant for *PER3*	0.000, 4.000	1.204, 1.762	1.702, 1.730	1.457	1.541 ± 0.028
**73. umRo**	Degradation rate constant for *CRY1*	0.202, 0.605	0.402, 0.600	0.570, 0.581	0.462	0.442 ± 0.007
**74. umRt**	Degradation rate constant for *CRY2*	0.228, 0.683	0.488, 0.683	0.507, 0.517	0.603	0.596 ± 0.010
**75. umB**	Degradation rate constant for *BMAL*	0.398, 1.193	0.814, 1.178	1.109, 1.127	0.917	0.926 ± 0.018
**76. umNp**	Degradation rate constant for *NPAS2*	0.185, 0.554	0.322, 0.536	0.449, 0.472	0.460	0.471 ± 0.011
**77. umRev**	Degradation rate constant for *REV-ERBs*	0.755, 2.265	1.436, 2.261	2.134, 2.175	1.978	1.729 ± 0.033
**78. upuo**	Degradation rate constant for unphosphorylated PER1	0.035, 0.105	0.040, 0.059	0.044, 0.046	0.045	0.046 ± 0.001
**79. upo**	Degradation rate constant for CKI-phosphorylated PER1	1.769, 5.306	2.218, 3.099	2.800, 2.886	2.384	2.610 ± 0.057
80. uput	Degradation rate constant for unphosphorylated PER2	0.000, 4.000	2.104, 3.827	2.167, 2.266	3.005	2.936 ± 0.077
81. uptc	Degradation rate constant for CKI-phosphorylated PER2	0.000, 4.000	2.667, 3.964	3.734, 3.838	3.811	3.593 ± 0.060
82. uptg	Degradation rate constant for GSK3β-phosphorylated PER2	0.000, 4.000	0.885, 1.462	1.096, 1.153	1.125	1.041 ± 0.024
83. uptcg	Degradation rate constant for CKI- and GSK3β-phosphorylated PER2	0.000, 4.000	2.603, 3.889	2.862, 2.991	3.719	3.244 ± 0.077
84. upuh	Degradation rate constant for unphosphorylated PER3	0.000, 4.000	2.431, 4.000	3.772, 3.921	3.473	3.297 ± 0.088
85. uph	Degradation rate constant for CKI-phosphorylated PER3	0.000, 4.000	0.945, 1.754	1.316, 1.383	1.349	1.386 ± 0.048
86. upuoh	Degradation rate constant for unphosphorylated PER1-PER3	0.000, 4.000	0.251, 0.466	0.301, 0.322	0.359	0.350 ± 0.012
87. upoh	Degradation rate constant for CKI-phosphorylated PER1-PER3	0.000, 4.000	0.995, 1.404	1.109, 1.140	1.080	1.174 ± 0.014

**88. uro**	Degradation rate constant for CRY1	0.087, 0.262	0.200, 0.253	0.247, 0.249	0.248	0.232 ± 0.003
**89. urt**	Degradation rate constant for CRY2	0.241, 0.723	0.447, 0.606	0.573, 0.581	0.580	0.554 ± 0.009
**90. ub**	Degradation rate constant for BMAL	0.009, 0.028	0.017, 0.028	0.018, 0.019	0.024	0.060 ± 0.012
**91. uc**	Degradation rate constant for CLOCK/NPAS2	0.013, 0.038	0.021, 0.032	0.027, 0.028	0.029	0.028 ± 0.000
**92. ubc**	Degradation rate constant for BMAL-CLOCK/NPAS2	0.174, 0.523	0.187, 0.271	0.198, 0.202	0.215	0.235 ± 0.005
**93. urev**	Degradation rate constant for unphosphorylated REV-ERBs	0.824, 2.473	1.409, 1.871	1.403, 1.475	1.439	1.432 ± 0.023
**94. uprev**	Degradation rate constant for GSK3β-phosphorylated REV-ERBs	0.259, 0.776	0.401, 0.674	0.474, 0.488	0.518	0.520 ± 0.015
**95. Nf**	Ratio of cytoplasmic to nuclear compartment volume	1.675, 5.026	1.692, 2.224	1.806, 1.832	1.711	1.880 ± 0.032
96. zhangsnpu	Increase PER3 degradation rate	1.000, 20.000	9.336, 12.619	9.464, 9.949	9.707	10.613 ± 0.263
97. zhangsnpa	Decrease PER1 to PER3 binding rate	0.000, 1.000	0.211, 0.392	0.294, 0.309	0.302	0.287 ± 0.007
98. per34h	Decrease PER1-PER3 complex phosphorylation rate	0.000, 1.000	0.292, 0.439	0.406, 0.427	0.417	0.364 ± 0.009
99. per35h	Increase PER1-PER3 complex phosphorylation rate	1.000, 20.000	7.495, 10.998	8.248, 8.671	8.460	8.707 ± 0.170
100.hidasnpa	Decrease PER3 to CKI binding rate	0.000, 1.000	0.225, 0.335	0.313, 0.329	0.321	0.288 ± 0.007

Table S3. Description of model variables.

Symbol	Species Name
**GR**	probability of *CRY1* E-box repression
**G**	probability of *CRY1* E-box activation
**GpoR**	probability of *PER1* E-box repression
**Gpo**	probability of *PER1* E-box activation
**GptR**	probability of *PER2* E-box repression
**Gpt**	probability of *PER2* E-box activation
GphR	probability of *PER3* E-box repression
Gph	probability of *PER3* E-box activation
**GrR**	probability of *REV-ERBs* E-box repression
**Gr**	probability of *REV-ERBs* E-box activation
**GcR**	probability of *CRY2* E-box repression
**Gc**	probability of *CRY2* E-box activation
**GBR**	probability of *CRY1* RORE repression
**GB**	probability of *CRY1* RORE activation
**GBRn**	probability of *NPAS2* RORE activation
**GBn**	probability of *NPAS2* RORE repression
**GBRb**	probability of *BMAL* RORE repression
**GBb**	probability of *BMAL* RORE activation
**MnPo**	concentration of *Per1 mRNA* in the nucleus
**McPo**	concentration of *Per1 mRNA* in the cytoplasm
**MnPt**	concentration of *Per2 mRNA* in the nucleus
**McPt**	concentration of *Per2 mRNA* in the cytoplasm
MnPh	concentration of *Per3 mRNA* in the nucleus
McPh	concentration of *Per3 mRNA* in the cytoplasm
**MnRt**	concentration of *Cry2 mRNA* in the nucleus
**McRt**	concentration of *Cry2 mRNA* in the cytoplasm
**MnRev**	concentration of *REV-ERB*s mRNA in the nucleus
**McRev**	concentration of *REV-ERB*s mRNA in the cytoplasm
**MnRo**	concentration of *Cry1 mRNA* in the nucleus
**McRo**	concentration of *Cry1 mRNA* in the cytoplasm
**MnB**	concentration of *Bmal1 mRNA* in the nucleus
**McB**	concentration of *Bmal1 mRNA* in the cytoplasm
**MnNp**	concentration of *Npas2 mRNA* in the nucleus
**McNp**	concentration of *Npas2 mRNA* in the cytoplasm
**B**	concentration of BMAL in the cytoplasm
**Cl**	concentration of CLOCK/NPAS2 in the cytoplasm
**cyrev**	concentration of unphosphorylated REV-ERBs in the cytoplasm
**revn**	concentration of unphosphorylated REV-ERBs in the nucleus
**cyrevg**	concentration of unphosphorylated REV-ERBs-GSK3β in the cytoplasm
**revng**	concentration of unphosphorylated REV-ERBs-GSK3β in the nucleus
**cyrevgp**	concentration of phosphorylatd REV-ERBs-GSK3β in the cytoplasm
**revngp**	concentration of phosphorylated REV-ERBs-GSK3β in the nucleus
**cyrevp**	concentration of phosphorylated REV-ERBs in the cytoplasm
**revnp**	concentration of phosphorylated REV-ERBs in the nucleus
**gto**	activity of GSK3β

Most proteins and complexes in the model are written using the guidelines provided below as follows: x[PER][CRY][Kinase][Location][BMAL-CLOCK/NPAS2]. For instance, x62000 would be CKI- and GSK3β-phosphorylated PER2, bound to CRY2, located in the cytoplasm of the cell. Meanwhile, x80100 would represent a PER1-PER3-CKI complex located in the cytoplasm of the cell, while x00110 would represent nuclear CKI.

**Index**	**PER**	**CRY**	**Kinase**	**Location**	**BMAL-CLOCK/NPAS2**
**0**	no PER bound	no CRY bound	no kinase bound	cytoplasm	no BMAL-CLOCK/NPAS2 bound
**1**	PER1	CRY1	CKI	nucleus	Phosphorylated BMAL-CLOCK/NPAS2
**2**	PER1 phosphorylated by CKI	CRY2	GSK3β		*Unphosphorylated BMAL-CLOCK/NPAS2*
**3**	PER2		CKI & GSK3β		
**4**	PER2 phosphorylated by CKI				
**5**	PER2 phosphorylated by GSK3β				
**6**	PER2 phosphorylated by CKI & GSK3β				
7	*PER3*				
8	*PER1-PER3*				
9	*PER1-PER3 phosphorylated by CKI*				
a	*PER3 phosphorylated by CKI*				

Table S4. Model equations.

1.	GR_dot = -(unbin*GR) +bin*(1-G-GR) *(x01011+x02011);
2.	G_dot = -(unbin*G) +bin*(1-G-GR) *x00011;
3.	GpoR_dot = -(unbinpo*GpoR) +binpo*(1-Gpo-GpoR) *(x01011+x02011);
4.	Gpo_dot = -(unbinpo*Gpo) +binpo*(1-Gpo-GpoR) *x00011;
5.	GptR_dot = -(unbinpt*GptR) +binpt*(1-Gpt-GptR) *(x01011+x02011);
6.	Gpt_dot = -(unbinpt*Gpt) +binpt*(1-Gpt-GptR) *x00011;
7.	GphR_dot = (-(unbinph*GphR) +binph*(1-Gph-GphR) *(x01011+x02011));
8.	Gph_dot = (-(unbinph*Gph) +binph*(1-Gph-GphR) *x00011);
9.	GrR_dot = -(unbinr*GrR) +binr*(1-Gr-GrR) *(x01011+x02011);
10.	Gr_dot = -(unbinr*Gr) +binr*(1-Gr-GrR) *x00011;
11.	GcR_dot = -(unbinc*GcR) +binc*(1-Gc-GcR) *(x01011+x02011);
12.	Gc_dot = -(unbinc*Gc) +binc*(1-Gc-GcR) *x00011;
13.	GBR_dot = -(unbinrev*GBR) +binrev*GB*(revn+revng+revngp+revnp);
14.	GB_dot = unbinrev*GBR-binrev*GB*(revn+revng+revngp+revnp);
15.	GBRn_dot = -(unbinrevn*GBRn) +binrevn*GBn*(revn+revng+revngp+revnp);
16.	GBn_dot = unbinrevn*GBRn-binrevn*GBn*(revn+revng+revngp+revnp);
17.	GBRb_dot = -(unbinrevb*GBRb) +binrevb*GBb*(revn+revng+revngp+revnp);
18.	GBb_dot = unbinrevb*GBRb-binrevb*GBb*(revn+revng+revngp+revnp);
19.	MnPo_dot = trPo*Gpo-tmc*MnPo-umPo*MnPo;
20.	McPo_dot = -(umPo*McPo) +tmc*MnPo;
21.	MnPt_dot = trPt*Gpt-tmc*MnPt-umPt*MnPt;
22.	McPt_dot = -(umPt*McPt) +tmc*MnPt;
23.	MnPh_dot = trPh*Gph-tmc*MnPh-umPh*MnPh;
24.	McPh_dot = -(umPh*McPh) +tmc*MnPh;
25.	MnRt_dot = trRt*Gc-tmc*MnRt-umRt*MnRt;
26.	McRt_dot = -(umRt*McRt) +tmc*MnRt;
27.	MnRev_dot = -(tmcrev*MnRev)-umRev*MnRev+trRev*Gr*x00011;
28.	McRev_dot = -(umRev*McRev) +tmcrev*MnRev;
29.	MnRo_dot = trRo*G*GB-tmc*MnRo-umRo*MnRo;
30.	McRo_dot = -(umRo*McRo) +tmc*MnRo;
31.	MnB_dot = trB*GBb-tmc*MnB-umB*MnB;
32.	McB_dot = -(umB*McB) +tmc*MnB;
33.	MnNp_dot = trNp*GBn-tmc*MnNp-umNp*MnNp;
34.	McNp_dot = -(umNp*McNp) +tmc*MnNp;
35.	B_dot = -(ub*B) +uncbin*x00002-cbin*B*Cl+tlb*McB;
36.	Cl_dot = tlc+uncbin*x00002-uc*Cl-cbin*B*Cl+tlnp*McNp;
37.	cyrev_dot = -((nlrev+urev) *cyrev) +dg*cyrevg+tlrev*McRev+nerev*revn-ag*cyrev*x00200;
38.	revn_dot = nlrev*cyrev+(-nerev-urev) *revn+dg*revng-ag*Nf*revn*x00210;
39.	cyrevg_dot = -(cyrevg*(dg+nlrev+urev+gto)) +nerev*revng+ag*cyrev*x00200;
40.	revng_dot = nlrev*cyrevg-(dg+nerev+urev+gto) *revng+ag*Nf*revn*x00210;
41.	cyrevgp_dot = -((dg+nlrev+uprev) *cyrevgp) +cyrevg*gto+nerev*revngp;
42.	revngp_dot = nlrev*cyrevgp+gto*revng-(dg+nerev+uprev) *revngp;
43.	cyrevp_dot = dg*cyrevgp-(nlrev+uprev) *cyrevp+nerev*revnp;
44.	revnp_dot = nlrev*cyrevp+dg*revngp-(nerev+uprev)*revnp;
45.	gto_dot = trgto*G*GB-ugto*gto;
46.	x00001_dot = phos*x00002-nlbc*x00001-ubc*x00001;
47.	x00002_dot = -(phos*x00002)-ubc*x00002-uncbin*x00002+cbin*B*Cl-bincryb*x00002*(x01000+x02000)+unbincryb*(x01002+x02002);
48.	x00011_dot = nlbc*x00001-ubc*x00011+uro*x01011-cbbin*Nf*x00011*(x01010+x02010)+urt*x02011+uncbbin*(x01011+x02011)+uptg*(x50011+x50111+x50211+x50311)+upo*(x20011+x20111)+uptc*(x40011+x40111+x40211+x40311)+uptcg*(x60011+x60111+x60211+x60311)+upoh*(x90011+x90111)+uph*xa0011-bbin*Nf*x00011*(x20010+x20110+x21010+x21110+x22010+x22110+x40010+x40110+x40210+x40310+x41010+x41110+x41210+x41310+x42010+x42110+x42210+x42310+x50010+x50110+x50210+x50310+x51010+x51110+x51210+x51310+x52010+x52110+x52210+x52310+x60010+x60110+x60210+x60310+x61010+x61110+x61210+x61310+x62010+x62110+x62210+x62310+x90010+x90110+xa0010+xa1010+xa2010)+unbbin*(x20011+x20111+x21011+x21111+x22011+x22111+x40011+x40111+x40211+x40311+x41011+x41111+x41211+x41311+x42011+x42111+x42211+x42311+x50011+x50111+x50211+x50311+x51011+x51111+x51211+x51311+x52011+x52111+x52211+x52311+x60011+x60111+x60211+x60311+x61011+x61111+x61211+x61311+x62011+x62111+x62211+x62311+x90011+x90111+xa0011+xa1011+xa2011);

49.	x00100_dot = lne*x00110+upuo*x10100+uput*(x30100+x30300)+uptg*(x50100+x50300)+upuoh*x80100+upo*x20100+uptc*(x40100+x40300)+uptcg*(x60100+x60300)+upoh*x90100-ac*x00100*(x10000+x20000+x21000+x22000+x30000+x40000+x41000+x42000+x50000+x51000+x52000+x60000+x61000+x62000)-acph*(x80000+x90000)+dc*(x10100+x20100+x21100+x22100+x30100+x40100+x41100+x42100+x50100+x51100+x52100+x60100+x61100+x62100+x80100+x90100)-ac*x00100*(x30200+x40200+x41200+x42200+x50200+x51200+x52200+x60200+x61200+x62200)+dc*(x30300+x40300+x41300+x42300+x50300+x51300+x52300+x60300+x61300+x62300);
50.	x00110_dot = -(lne*x00110)+uptg*(x50110+x50111+x50310+x50311)+upo*(x20110+x20111)+uptc*(x40110+x40111+x40310+x40311)+uptcg*(x60110+x60111+x60310+x60311)+upoh*(x90110+x90111)-ac*Nf*x00110*(x20010+x21010+x22010+x40010+x41010+x42010+x50010+x51010+x52010+x60010+x61010+x62010)-acph*x90010-ac*Nf*x00110*(x20011+x21011+x22011+x40011+x41011+x42011+x50011+x51011+x52011+x60011+x61011+x62011-acph*x90011+dc*(x20110+x21110+x22110+x40110+x41110+x42110+x50110+x51110+x52110+x60110+x61110+x62110+x90110)+dc*(x20111+x21111+x22111+x40111+x41111+x42111+x50111+x51111+x52111+x60111+x61111+x62111+x90111)-ac*Nf*x00110*(x40210+x41210+x42210+x50210+x51210+x52210+x60210+x61210+x62210)-ac*Nf*x00110*(x40211+x41211+x42211+x50211+x51211+x52211+x60211+x61211+x62211)+dc*(x40310+x41310+x42310+x50310+x51310+x52310+x60310+x61310+x62310)+dc*(x40311+x41311+x42311+x50311+x51311+x52311+x60311+x61311+x62311);
51.	x00200_dot = dg*cyrevg+urev*cyrevg+dg*cyrevgp+uprev*cyrevgp-ag*cyrev*x00200+lne*x00210+uput*(x30200+x30300)+uptg*(x50200+x50300)+uptc*(x40200+x40300)+uptcg*(x60200+x60300)-agp*x00200*(x30000+x30100+x40000+x40100+x41000+x41100+x42000+x42100+x50000+x50100+x51000+x51100+x52000+x52100+x60000+x60100+x61000+x61100+x62000+x62100)+dg*(x30200+x30300+x40200+x40300+x41200+x41300+x42200+x42300+x50200+x50300+x51200+x51300+x52200+x52300+x60200+x60300+x61200+x61300+x62200+x62300);
52.	x00210_dot = dg*revng+urev*revng+dg*revngp+uprev*revngp-lne*x00210-ag*Nf*revn*x00210+uptg*(x50210+x50211+x50310+x50311)+uptc*(x40210+x40211+x40310+x40311)+uptcg*(x60210+x60211+x60310+x60311)-agp*Nf*x00210*(x40010+x40011+x40110+x40111+x41010+x41011+x41110+x41111+x42010+x42011+x42110+x42111+x50010+x50011+x50110+x50111+x51010+x51011+x51110+x51111+x52010+x52011+x52110+x52111+x60010+x60011+x60110+x60111+x61010+x61011+x61110+x61111+x62010+x62011+x62110+x62111)+dg*(x40210+x40211+x40310+x40311+x41210+x41211+x41310+x41311+x42210+x42211+x42310+x42311+x50210+x50211+x50310+x50311+x51210+x51211+x51310+x51311+x52210+x52211+x52310+x52311+x60210+x60211+x60310+x60311+x61210+x61211+x61310+x61311+x62210+x62211+x62310+x62311);
53.	x01000_dot = tlro*McRo-bincryb*x00002*x01000+unbincryb*x01002-uro*x01000-ar*x01000*(x20000+x20100+x40000+x40100+x40200+x40300+x50000+x50100+x50200+x50300+x60000+x60100+x60200+x60300)+dr*(x21000+x21100+x41000+x41100+x41200+x41300+x51000+x51100+x51200+x51300+x61000+x61100+x61200+x61300);
54.	x01002_dot = bincryb*x00002*x01000-unbincryb*x01002;
55.	x01010_dot = -(uro*x01010)-cbbin*Nf*x00011*x01010+uncbbin*x01011-ar*Nf*x01010*(x20010+x20110+x40010+x40110+x40210+x40310+x50010+x50110+x50210+x50310+x60010+x60110+x60210+x60310)-arph*Nf*xa0010-ar*Nf*x01010*(x20011+x20111+x40011+x40111+x40211+x40311+x50011+x50111+x50211+x50311+x60011+x60111+x60211+x60311)-ar*Nf*xa0011+dr*(x21010+x21110+x41010+x41110+x41210+x41310+x51010+x51110+x51210+x51310+x61010+x61110+x61210+x61310)+drph*xa1010+dr*(x21011+x21111+x41011+x41111+x41211+x41311+x51011+x51111+x51211+x51311+x61011+x61111+x61211+x61311)+drph*xa1011;
56.	x01011_dot = cbbin*Nf*x00011*x01010-uncbbin*x01011-uro*x01011-ar*Nf*x01011*(x20010+x20110+x40010+x40110+x40210+x40310+x50010+x50110+x50210+x50310+x60010+x60110+x60210+x60310)-arph*Nf*xa0010+dr*(x21011+x21111+x41011+x41111+x41211+x41311+x51011+x51111+x51211+x51311+x61011+x61111+x61211+x61311)+drph*xa1011;
57.	x02000_dot = tlrt*McRt-bincryb*x00002*x02000+unbincryb*x02002-urt*x02000-ar*x02000*(x20000+x20100+x40000+x40100+x40200+x40300+x50000+x50100+x50200+x50300+x60000+x60100+x60200+x60300)+dr*(x22000+x22100+x42000+x42100+x42200+x42300+x52000+x52100+x52200+x52300+x62000+x62100+x62200+x62300);
58.	x02002_dot = bincryb*x00002*x02000-unbincryb*x02002;

59.	x02010_dot = -(urt*x02010)-cbbin*Nf*x00011*x02010+uncbbin*x02011-ar*Nf*x02010*(x20010+x20110+x40010+x40110+x40210+x40310+x50010+x50110+x50210+x50310+x60010+x60110+x60210+x60310)-arph*Nf*xa0010-ar*Nf*x02010*(x20011+x20111+x40011+x40111+x40211+x40311+x50011+x50111+x50211+x50311+x60011+x60111+x60211+x60311)-arph*Nf*xa0011+dr*(x22010+x22110+x42010+x42110+x42210+x42310+x52010+x52110+x52210+x52310+x62010+x62110+x62210+x62310)+drph*xa2010+dr*(x22011+x22111+x42011+x42111+x42211+x42311+x52011+x52111+x52211+x52311+x62011+x62111+x62211+x62311)+drph*xa2011;
60.	x02011_dot = cbbin*Nf*x00011*x02010-uncbbin*x02011-urt*x02011-ar*Nf*x02011*(x20010+x20110+x40010+x40110+x40210+x40310+x50010+x50110+x50210+x50310+x60010+x60110+x60210+x60310)-arph*Nf*xa0010+dr*(x22011+x22111+x42011+x42111+x42211+x42311+x52011+x52111+x52211+x52311+x62011+x62111+x62211+x62311)+drph*xa2011;
61.	x10000_dot = tlpo*McPo-upuo*x10000-ac*x00100*x10000+dc*x10100-an*x70000*x10000+dn*x80000;
62.	x10100_dot = ac*x00100*x10000-dc*x10100-hoo*x10100-upuo*x10100;
63.	x20000_dot = -(nl*x20000)-upo*x20000-ac*x00100*x20000-ar*(x01000+x02000)*x20000+ne*x20010+dc*x20100+dr*(x21000+x22000)-an*xa0000*x20000+dn*x90000;
64.	x20010_dot = nl*x20000-ne*x20010-upo*x20010-bbin*Nf*x00011*x20010-ac*Nf*x00110*x20010-ar*Nf*(x01010+x02010)*x20010-ar*Nf*(x01011+x02011)*x20010+ubc*x20011+unbbin*x20011+dc*x20110+dr*(x21010+x22010)+dr*(x21011+x22011)-an*Nf*xa0010*x20010+dn*x90010-an*Nf*xa0011*x20010+dn*x90011;
65.	x20011_dot = bbin*Nf*x00011*x20010-ubc*x20011-unbbin*x20011-upo*x20011-ac*Nf*x00110*x20011-ar*Nf*(x01010+x02010)*x20011+dc*x20111+dr*(x21011+x22011)-an*Nf*x20011*xa0010+dn*x90011;
66.	x20100_dot = hoo*x10100+ac*x00100*x20000-dc*x20100-nl*x20100-upo*x20100-ar*(x01000+x02000)*x20100+ne*x20110+dr*(x21100+x22100);
67.	x20110_dot = ac*Nf*x00110*x20010+nl*x20100-dc*x20110-ne*x20110-upo*x20110-bbin*Nf*x00011*x20110-ar*Nf*(x01010+x02010)*x20110-ar*Nf*(x01011+x02011)*x20110+ubc*x20111+unbbin*x20111+dr*(x21110+x22110)+dr*(x21111+x22111);
68.	x20111_dot = ac*Nf*x00110*x20011+bbin*Nf*x00011*x20110-dc*x20111-ubc*x20111-unbbin*x20111-upo*x20111-ar*Nf*(x01010+x02010)*x20111+dr*(x21111+x22111);
69.	x21000_dot = ar*x01000*x20000-dr*x21000-nl*x21000-ac*x00100*x21000+ne*x21010+dc*x21100;
70.	x21010_dot = ar*Nf*x01010*x20010+nl*x21000-dr*x21010-ne*x21010-bbin*Nf*x00011*x21010-ac*Nf*x00110*x21010+unbbin*x21011+dc*x21110;
71.	x21011_dot = ar*Nf*x01011*x20010+ar*Nf*x01010*x20011+bbin*Nf*x00011*x21010-2*dr*x21011-unbbin*x21011-ac*Nf*x00110*x21011+dc*x21111;
72.	x21100_dot = ar*x01000*x20100+ac*x00100*x21000-dc*x21100-dr*x21100-nl*x21100+ne*x21110;
73.	x21110_dot = ar*Nf*x01010*x20110+ac*Nf*x00110*x21010+nl*x21100-dc*x21110-dr*x21110-ne*x21110-bbin*Nf*x00011*x21110+unbbin*x21111;
74.	x21111_dot = ar*Nf*x01011*x20110+ar*Nf*x01010*x20111+ac*Nf*x00110*x21011+bbin*Nf*x00011*x21110-dc*x21111-2*dr*x21111-unbbin*x21111;
75.	x22000_dot = ar*x02000*x20000-dr*x22000-nl*x22000-ac*x00100*x22000+ne*x22010+dc*x22100;
76.	x22010_dot = ar*Nf*x02010*x20010+nl*x22000-dr*x22010-ne*x22010-bbin*Nf*x00011*x22010-ac*Nf*x00110*x22010+unbbin*x22011+dc*x22110;
77.	x22011_dot = ar*Nf*x02011*x20010+ar*Nf*x02010*x20011+bbin*Nf*x00011*x22010-2*dr*x22011-unbbin*x22011-ac*Nf*x00110*x22011+dc*x22111;
78.	x22100_dot = ar*x02000*x20100+ac*x00100*x22000-dc*x22100-dr*x22100-nl*x22100+ne*x22110;
79.	x22110_dot = ar*Nf*x02010*x20110+ac*Nf*x00110*x22010+nl*x22100-dc*x22110-dr*x22110-ne*x22110-bbin*Nf*x00011*x22110+unbbin*x22111;
80.	x22111_dot = ar*Nf*x02011*x20110+ar*Nf*x02010*x20111+ac*Nf*x00110*x22011+bbin*Nf*x00011*x22110-dc*x22111-2*dr*x22111-unbbin*x22111;
81.	x30000_dot = tlpt*McPt-uput*x30000-ac*x00100*x30000-agp*x00200*x30000+dc*x30100+dg*x30200;
82.	x30100_dot = ac*x00100*x30000-dc*x30100-hto*x30100-uput*x30100-agp*x00200*x30100+dg*x30300;
83.	x30200_dot = agp*x00200*x30000-dg*x30200-uput*x30200-gto*x30200-ac*x00100*x30200+dc*x30300;
84.	x30300_dot = agp*x00200*x30100+ac*x00100*x30200-dc*x30300-dg*x30300-hto*x30300-uput*x30300-gto*x30300;
85.	x40000_dot = -(nl*x40000)-uptc*x40000-ac*x00100*x40000-agp*x00200*x40000-ar*(x01000+x02000)*x40000+ne*x40010+dc*x40100+dg*x40200+dr*(x41000+x42000);
86.	x40010_dot = nl*x40000-ne*x40010-uptc*x40010-bbin*Nf*x00011*x40010-ac*Nf*x00110*x40010-agp*Nf*x00210*x40010-ar*Nf*(x01010+x02010)*x40010-ar*Nf*(x01011+x02011)*x40010+ubc*x40011+unbbin*x40011+dc*x40110+dg*x40210+dr*(x41010+x42010)+dr*(x41011+x42011);
87.	x40011_dot = bbin*Nf*x00011*x40010-ubc*x40011-unbbin*x40011-uptc*x40011-ac*Nf*x00110*x40011-agp*Nf*x00210*x40011-ar*Nf*(x01010+x02010)*x40011+dc*x40111+dg*x40211+dr*(x41011+x42011);

88. x40100_dot = hto*x30100+ac*x00100*x40000-dc*x40100-nl*x40100-uptc*x40100-agp*x00200*x40100-ar*(x01000+x02000)*x40100+ne*x40110+dg*x40300+dr*(x41100+x42100);
89. x40110_dot = ac*Nf*x00110*x40010+nl*x40100-dc*x40110-ne*x40110-uptc*x40110-bbin*Nf*x00011*x40110-agp*Nf*x00210*x40110-ar*Nf*(x01010+x02010)*x40110-ar*Nf*(x01011+x02011)*x40110+ubc*x40111+unbbin*x40111+dg*x40310+dr*(x41110+x42110)+dr*(x41111+x42111);
90. x40111_dot = ac*Nf*x00110*x40011+bbin*Nf*x00011*x40110-dc*x40111-ubc*x40111-unbbin*x40111-uptc*x40111-agp*Nf*x00210*x40111-ar*Nf*(x01010+x02010)*x40111+dg*x40311+dr*(x41111+x42111);
91. x40200_dot = agp*x00200*x40000-dg*x40200-nl*x40200-uptc*x40200-gto*x40200-ac*x00100*x40200-ar*(x01000+x02000)*x40200+ne*x40210+dc*x40300+dr*(x41200+x42200);
92. x40210_dot = agp*Nf*x00210*x40010+nl*x40200-dg*x40210-ne*x40210-uptc*x40210-gto*x40210-bbin*Nf*x00011*x40210-ac*Nf*x00110*x40210-ar*Nf*(x01010+x02010)*x40210-ar*Nf*(x01011+x02011)*x40210+ubc*x40211+unbbin*x40211+dc*x40310+dr*(x41210+x42210)+dr*(x41211+x42211);
93. x40211_dot = agp*Nf*x00210*x40011+bbin*Nf*x00011*x40210-dg*x40211-ubc*x40211-unbbin*x40211-uptc*x40211-gto*x40211-ac*Nf*x00110*x40211-ar*Nf*(x01010+x02010)*x40211+dc*x40311+dr*(x41211+x42211);
94. x40300_dot = hto*x30300+agp*x00200*x40100+ac*x00100*x40200-dc*x40300-dg*x40300-nl*x40300-uptc*x40300-gto*x40300-ar*(x01000+x02000)*x40300+ne*x40310+dr*(x41300+x42300);
95. x40310_dot = agp*Nf*x00210*x40110+ac*Nf*x00110*x40210+nl*x40300-dc*x40310-dg*x40310-ne*x40310-uptc*x40310-gto*x40310-bbin*Nf*x00011*x40310-ar*Nf*(x01010+x02010)*x40310-ar*Nf*(x01011+x02011)*x40310+ubc*x40311+unbbin*x40311+dr*(x41310+x42310)+dr*(x41311+x42311);
96. x40311_dot = agp*Nf*x00210*x40111+ac*Nf*x00110*x40211+bbin*Nf*x00011*x40310-dc*x40311-dg*x40311-ubc*x40311-unbbin*x40311-uptc*x40311-gto*x40311-ar*Nf*(x01010+x02010)*x40311+dr*(x41311+x42311);
97. x41000_dot = ar*x01000*x40000-dr*x41000-nl*x41000-ac*x00100*x41000-agp*x00200*x41000+ne*x41010+dc*x41100+dg*x41200;
98. x41010_dot = ar*Nf*x01010*x40010+nl*x41000-dr*x41010-ne*x41010-bbin*Nf*x00011*x41010-ac*Nf*x00110*x41010-agp*Nf*x00210*x41010+unbbin*x41011+dc*x41110+dg*x41210;
99. x41011_dot = ar*Nf*x01011*x40010+ar*Nf*x01010*x40011+bbin*Nf*x00011*x41010-2*dr*x41011-unbbin*x41011-ac*Nf*x00110*x41011-agp*Nf*x00210*x41011+dc*x41111+dg*x41211;
100. x41100_dot = ar*x01000*x40100+ac*x00100*x41000-dc*x41100-dr*x41100-nl*x41100-agp*x00200*x41100+ne*x41110+dg*x41300;
101. x41110_dot = ar*Nf*x01010*x40110+ac*Nf*x00110*x41010+nl*x41100-dc*x41110-dr*x41110-ne*x41110-bbin*Nf*x00011*x41110-agp*Nf*x00210*x41110+unbbin*x41111+dg*x41310;
102. x41111_dot = ar*Nf*x01011*x40110+ar*Nf*x01010*x40111+ac*Nf*x00110*x41011+bbin*Nf*x00011*x41110-dc*x41111-2*dr*x41111-unbbin*x41111-agp*Nf*x00210*x41111+dg*x41311;
103. x41200_dot = ar*x01000*x40200+agp*x00200*x41000-dg*x41200-dr*x41200-nl*x41200-gto*x41200-ac*x00100*x41200+ne*x41210+dc*x41300;
104. x41210_dot = ar*Nf*x01010*x40210+agp*Nf*x00210*x41010+nl*x41200-dg*x41210-dr*x41210-ne*x41210-gto*x41210-bbin*Nf*x00011*x41210-ac*Nf*x00110*x41210+unbbin*x41211+dc*x41310;
105. x41211_dot = ar*Nf*x01011*x40210+ar*Nf*x01010*x40211+agp*Nf*x00210*x41011+bbin*Nf*x00011*x41210-dg*x41211-2*dr*x41211-unbbin*x41211-gto*x41211-ac*Nf*x00110*x41211+dc*x41311;
106. x41300_dot = ar*x01000*x40300+agp*x00200*x41100+ac*x00100*x41200-dc*x41300-dg*x41300-dr*x41300-nl*x41300-gto*x41300+ne*x41310;
107. x41310_dot = ar*Nf*x01010*x40310+agp*Nf*x00210*x41110+ac*Nf*x00110*x41210+nl*x41300-dc*x41310-dg*x41310-dr*x41310-ne*x41310-gto*x41310-bbin*Nf*x00011*x41310+unbbin*x41311;
108. x41311_dot = ar*Nf*x01011*x40310+ar*Nf*x01010*x40311+agp*Nf*x00210*x41111+ac*Nf*x00110*x41211+bbin*Nf*x00011*x41310-dc*x41311-dg*x41311-2*dr*x41311-unbbin*x41311-gto*x41311;
109. x42000_dot = ar*x02000*x40000-dr*x42000-nl*x42000-ac*x00100*x42000-agp*x00200*x42000+ne*x42010+dc*x42100+dg*x42200;
110. x42010_dot = ar*Nf*x02010*x40010+nl*x42000-dr*x42010-ne*x42010-bbin*Nf*x00011*x42010-ac*Nf*x00110*x42010-agp*Nf*x00210*x42010+unbbin*x42011+dc*x42110+dg*x42210;
111. x42011_dot = ar*Nf*x02011*x40010+ar*Nf*x02010*x40011+bbin*Nf*x00011*x42010-2*dr*x42011-unbbin*x42011-ac*Nf*x00110*x42011-agp*Nf*x00210*x42011+dc*x42111+dg*x42211;
112. x42100_dot = ar*x02000*x40100+ac*x00100*x42000-dc*x42100-dr*x42100-nl*x42100-agp*x00200*x42100+ne*x42110+dg*x42300;
113. x42110_dot = ar*Nf*x02010*x40110+ac*Nf*x00110*x42010+nl*x42100-dc*x42110-dr*x42110-ne*x42110-bbin*Nf*x00011*x42110-agp*Nf*x00210*x42110+unbbin*x42111+dg*x42310;
114. x42111_dot = ar*Nf*x02011*x40110+ar*Nf*x02010*x40111+ac*Nf*x00110*x42011+bbin*Nf*x00011*x42110-dc*x42111-2*dr*x42111-unbbin*x42111-agp*Nf*x00210*x42111+dg*x42311;
115. x42200_dot = ar*x02000*x40200+agp*x00200*x42000-dg*x42200-dr*x42200-nl*x42200-gto*x42200-ac*x00100*x42200+ne*x42210+dc*x42300;
116. x42210_dot = ar*Nf*x02010*x40210+agp*Nf*x00210*x42010+nl*x42200-dg*x42210-dr*x42210-ne*x42210-gto*x42210-bbin*Nf*x00011*x42210-ac*Nf*x00110*x42210+unbbin*x42211+dc*x42310;

117. x42211_dot = ar*Nf*x02011*x40210+ar*Nf*x02010*x40211+agp*Nf*x00210*x42011+bbin*Nf*x00011*x42210-dg*x42211-2*dr*x42211-unbbin*x42211-gto*x42211-ac*Nf*x00110*x42211+dc*x42311;
118. x42300_dot = ar*x02000*x40300+agp*x00200*x42100+ac*x00100*x42200-dc*x42300-dg*x42300-dr*x42300-nl*x42300-gto*x42300+ne*x42310;
119. x42310_dot = ar*Nf*x02010*x40310+agp*Nf*x00210*x42110+ac*Nf*x00110*x42210+nl*x42300-dc*x42310-dg*x42310-dr*x42310-ne*x42310-gto*x42310-bbin*Nf*x00011*x42310+unbbin*x42311;
120. x42311_dot = ar*Nf*x02011*x40310+ar*Nf*x02010*x40311+agp*Nf*x00210*x42111+ac*Nf*x00110*x42211+bbin*Nf*x00011*x42310-dc*x42311-dg*x42311-2*dr*x42311-unbbin*x42311-gto*x42311;
121. x50000_dot = -(nl*x50000)-uptg*x50000-ac*x00100*x50000-agp*x00200*x50000-ar*(x01000+x02000)*x50000+ne*x50010+dc*x50100+dg*x50200+dr*(x51000+x52000);
122. x50010_dot = nl*x50000-ne*x50010-uptg*x50010-bbin*Nf*x00011*x50010-ac*Nf*x00110*x50010-agp*Nf*x00210*x50010-ar*Nf*(x01010+x02010)*x50010-ar*Nf*(x01011+x02011)*x50010+ubc*x50011+unbbin*x50011+dc*x50110+dg*x50210+dr*(x51010+x52010)+dr*(x51011+x52011);
123. x50011_dot = bbin*Nf*x00011*x50010-ubc*x50011-unbbin*x50011-uptg*x50011-ac*Nf*x00110*x50011-agp*Nf*x00210*x50011-ar*Nf*(x01010+x02010)*x50011+dc*x50111+dg*x50211+dr*(x51011+x52011);
124. x50100_dot = ac*x00100*x50000-dc*x50100-hto*x50100-nl*x50100-uptg*x50100-agp*x00200*x50100-ar*(x01000+x02000)*x50100+ne*x50110+dg*x50300+dr*(x51100+x52100);
125. x50110_dot = ac*Nf*x00110*x50010+nl*x50100-dc*x50110-hto*x50110-ne*x50110-uptg*x50110-bbin*Nf*x00011*x50110-agp*Nf*x00210*x50110-ar*Nf*(x01010+x02010)*x50110-ar*Nf*(x01011+x02011)*x50110+ubc*x50111+unbbin*x50111+dg*x50310+dr*(x51110+x52110)+dr*(x51111+x52111);
126. x50111_dot = ac*Nf*x00110*x50011+bbin*Nf*x00011*x50110-dc*x50111-hto*x50111-ubc*x50111-unbbin*x50111-uptg*x50111-agp*Nf*x00210*x50111-ar*Nf*(x01010+x02010)*x50111+dg*x50311+dr*(x51111+x52111);
127. x50200_dot = gto*x30200+agp*x00200*x50000-dg*x50200-nl*x50200-uptg*x50200-ac*x00100*x50200-ar*(x01000+x02000)*x50200+ne*x50210+dc*x50300+dr*(x51200+x52200);
128. x50210_dot = agp*Nf*x00210*x50010+nl*x50200-dg*x50210-ne*x50210-uptg*x50210-bbin*Nf*x00011*x50210-ac*Nf*x00110*x50210-ar*Nf*(x01010+x02010)*x50210-ar*Nf*(x01011+x02011)*x50210+ubc*x50211+unbbin*x50211+dc*x50310+dr*(x51210+x52210)+dr*(x51211+x52211);
129. x50211_dot = agp*Nf*x00210*x50011+bbin*Nf*x00011*x50210-dg*x50211-ubc*x50211-unbbin*x50211-uptg*x50211-ac*Nf*x00110*x50211-ar*Nf*(x01010+x02010)*x50211+dc*x50311+dr*(x51211+x52211);
130. x50300_dot = gto*x30300+agp*x00200*x50100+ac*x00100*x50200-dc*x50300-dg*x50300-hto*x50300-nl*x50300-uptg*x50300-ar*(x01000+x02000)*x50300+ne*x50310+dr*(x51300+x52300);
131. x50310_dot = agp*Nf*x00210*x50110+ac*Nf*x00110*x50210+nl*x50300-dc*x50310-dg*x50310-hto*x50310-ne*x50310-uptg*x50310-bbin*Nf*x00011*x50310-ar*Nf*(x01010+x02010)*x50310-ar*Nf*(x01011+x02011)*x50310+ubc*x50311+unbbin*x50311+dr*(x51310+x52310)+dr*(x51311+x52311);
132. x50311_dot = agp*Nf*x00210*x50111+ac*Nf*x00110*x50211+bbin*Nf*x00011*x50310-dc*x50311-dg*x50311-hto*x50311-ubc*x50311-unbbin*x50311-uptg*x50311-ar*Nf*(x01010+x02010)*x50311+dr*(x51311+x52311);
133. x51000_dot = ar*x01000*x50000-dr*x51000-nl*x51000-ac*x00100*x51000-agp*x00200*x51000+ne*x51010+dc*x51100+dg*x51200;
134. x51010_dot = ar*Nf*x01010*x50010+nl*x51000-dr*x51010-ne*x51010-bbin*Nf*x00011*x51010-ac*Nf*x00110*x51010-agp*Nf*x00210*x51010+unbbin*x51011+dc*x51110+dg*x51210;
135. x51011_dot = ar*Nf*x01011*x50010+ar*Nf*x01010*x50011+bbin*Nf*x00011*x51010-2*dr*x51011-unbbin*x51011-ac*Nf*x00110*x51011-agp*Nf*x00210*x51011+dc*x51111+dg*x51211;
136. x51100_dot = ar*x01000*x50100+ac*x00100*x51000-dc*x51100-dr*x51100-nl*x51100-agp*x00200*x51100+ne*x51110+dg*x51300;
137. x51110_dot = ar*Nf*x01010*x50110+ac*Nf*x00110*x51010+nl*x51100-dc*x51110-dr*x51110-ne*x51110-bbin*Nf*x00011*x51110-agp*Nf*x00210*x51110+unbbin*x51111+dg*x51310;
138. x51111_dot = ar*Nf*x01011*x50110+ar*Nf*x01010*x50111+ac*Nf*x00110*x51011+bbin*Nf*x00011*x51110-dc*x51111-2*dr*x51111-unbbin*x51111-agp*Nf*x00210*x51111+dg*x51311;
139. x51200_dot = ar*x01000*x50200+agp*x00200*x51000-dg*x51200-dr*x51200-nl*x51200-ac*x00100*x51200+ne*x51210+dc*x51300;
140. x51210_dot = ar*Nf*x01010*x50210+agp*Nf*x00210*x51010+nl*x51200-dg*x51210-dr*x51210-ne*x51210-bbin*Nf*x00011*x51210-ac*Nf*x00110*x51210+unbbin*x51211+dc*x51310;
141. x51211_dot = ar*Nf*x01011*x50210+ar*Nf*x01010*x50211+agp*Nf*x00210*x51011+bbin*Nf*x00011*x51210-dg*x51211-2*dr*x51211-unbbin*x51211-ac*Nf*x00110*x51211+dc*x51311;
142. x51300_dot = ar*x01000*x50300+agp*x00200*x51100+ac*x00100*x51200-dc*x51300-dg*x51300-dr*x51300-nl*x51300+ne*x51310;
143. x51310_dot = ar*Nf*x01010*x50310+agp*Nf*x00210*x51110+ac*Nf*x00110*x51210+nl*x51300-dc*x51310-dg*x51310-dr*x51310-ne*x51310-bbin*Nf*x00011*x51310+unbbin*x51311;
144. x51311_dot = ar*Nf*x01011*x50310+ar*Nf*x01010*x50311+agp*Nf*x00210*x51111+ac*Nf*x00110*x51211+bbin*Nf*x00011*x51310-dc*x51311-dg*x51311-2*dr*x51311-unbbin*x51311;

145. x52000_dot = ar*x02000*x50000-dr*x52000-nl*x52000-ac*x00100*x52000-agp*x00200*x52000+ne*x52010+dc*x52100+dg*x52200;
146. x52010_dot = ar*Nf*x02010*x50010+nl*x52000-dr*x52010-ne*x52010-bbin*Nf*x00011*x52010-ac*Nf*x00110*x52010-agp*Nf*x00210*x52010+unbbin*x52011+dc*x52110+dg*x52210;
147. x52011_dot = ar*Nf*x02011*x50010+ar*Nf*x02010*x50011+bbin*Nf*x00011*x52010-2*dr*x52011-unbbin*x52011-ac*Nf*x00110*x52011-agp*Nf*x00210*x52011+dc*x52111+dg*x52211;
148. x52100_dot = ar*x02000*x50100+ac*x00100*x52000-dc*x52100-dr*x52100-nl*x52100-agp*x00200*x52100+ne*x52110+dg*x52300;
149. x52110_dot = ar*Nf*x02010*x50110+ac*Nf*x00110*x52010+nl*x52100-dc*x52110-dr*x52110-ne*x52110-bbin*Nf*x00011*x52110-agp*Nf*x00210*x52110+unbbin*x52111+dg*x52310;
150. x52111_dot = ar*Nf*x02011*x50110+ar*Nf*x02010*x50111+ac*Nf*x00110*x52011+bbin*Nf*x00011*x52110-dc*x52111-2*dr*x52111-unbbin*x52111-agp*Nf*x00210*x52111+dg*x52311;
151. x52200_dot = ar*x02000*x50200+agp*x00200*x52000-dg*x52200-dr*x52200-nl*x52200-ac*x00100*x52200+ne*x52210+dc*x52300;
152. x52210_dot = ar*Nf*x02010*x50210+agp*Nf*x00210*x52010+nl*x52200-dg*x52210-dr*x52210-ne*x52210-bbin*Nf*x00011*x52210-ac*Nf*x00110*x52210+unbbin*x52211+dc*x52310;
153. x52211_dot = ar*Nf*x02011*x50210+ar*Nf*x02010*x50211+agp*Nf*x00210*x52011+bbin*Nf*x00011*x52210-dg*x52211-2*dr*x52211-unbbin*x52211-ac*Nf*x00110*x52211+dc*x52311;
154. x52300_dot = ar*x02000*x50300+agp*x00200*x52100+ac*x00100*x52200-dc*x52300-dg*x52300-dr*x52300-nl*x52300+ne*x52310;
155. x52310_dot = ar*Nf*x02010*x50310+agp*Nf*x00210*x52110+ac*Nf*x00110*x52210+nl*x52300-dc*x52310-dg*x52310-dr*x52310-ne*x52310-bbin*Nf*x00011*x52310+unbbin*x52311;
156. x52311_dot = ar*Nf*x02011*x50310+ar*Nf*x02010*x50311+agp*Nf*x00210*x52111+ac*Nf*x00110*x52211+bbin*Nf*x00011*x52310-dc*x52311-dg*x52311-2*dr*x52311-unbbin*x52311;
157. x60000_dot = -(nl*x60000)-uptcg*x60000-ac*x00100*x60000-agp*x00200*x60000-ar*(x01000+x02000)*x60000+ne*x60010+dc*x60100+dg*x60200+dr*(x61000+x62000);
158. x60010_dot = nl*x60000-ne*x60010-uptcg*x60010-bbin*Nf*x00011*x60010-ac*Nf*x00110*x60010-agp*Nf*x00210*x60010-ar*Nf*(x01010+x02010)*x60010-ar*Nf*(x01011+x02011)*x60010+ubc*x60011+unbbin*x60011+dc*x60110+dg*x60210+dr*(x61010+x62010)+dr*(x61011+x62011);
159. x60011_dot = bbin*Nf*x00011*x60010-ubc*x60011-unbbin*x60011-uptcg*x60011-ac*Nf*x00110*x60011-agp*Nf*x00210*x60011-ar*Nf*(x01010+x02010)*x60011+dc*x60111+dg*x60211+dr*(x61011+x62011);
160. x60100_dot = hto*x50100+ac*x00100*x60000-dc*x60100-nl*x60100-uptcg*x60100-agp*x00200*x60100-ar*(x01000+x02000)*x60100+ne*x60110+dg*x60300+dr*(x61100+x62100);
161. x60110_dot = hto*x50110+ac*Nf*x00110*x60010+nl*x60100-dc*x60110-ne*x60110-uptcg*x60110-bbin*Nf*x00011*x60110-agp*Nf*x00210*x60110-ar*Nf*(x01010+x02010)*x60110-ar*Nf*(x01011+x02011)*x60110+ubc*x60111+unbbin*x60111+dg*x60310+dr*(x61110+x62110)+dr*(x61111+x62111);
162. x60111_dot = hto*x50111+ac*Nf*x00110*x60011+bbin*Nf*x00011*x60110-dc*x60111-ubc*x60111-unbbin*x60111-uptcg*x60111-agp*Nf*x00210*x60111-ar*Nf*(x01010+x02010)*x60111+dg*x60311+dr*(x61111+x62111);
163. x60200_dot = gto*x40200+agp*x00200*x60000-dg*x60200-nl*x60200-uptcg*x60200-ac*x00100*x60200-ar*(x01000+x02000)*x60200+ne*x60210+dc*x60300+dr*(x61200+x62200);
164. x60210_dot = gto*x40210+agp*Nf*x00210*x60010+nl*x60200-dg*x60210-ne*x60210-uptcg*x60210-bbin*Nf*x00011*x60210-ac*Nf*x00110*x60210-ar*Nf*(x01010+x02010)*x60210-ar*Nf*(x01011+x02011)*x60210+ubc*x60211+unbbin*x60211+dc*x60310+dr*(x61210+x62210)+dr*(x61211+x62211);
165. x60211_dot = gto*x40211+agp*Nf*x00210*x60011+bbin*Nf*x00011*x60210-dg*x60211-ubc*x60211-unbbin*x60211-uptcg*x60211-ac*Nf*x00110*x60211-ar*Nf*(x01010+x02010)*x60211+dc*x60311+dr*(x61211+x62211);
166. x60300_dot = gto*x40300+hto*x50300+agp*x00200*x60100+ac*x00100*x60200-dc*x60300-dg*x60300-nl*x60300-uptcg*x60300-ar*(x01000+x02000)*x60300+ne*x60310+dr*(x61300+x62300);
167. x60310_dot = gto*x40310+hto*x50310+agp*Nf*x00210*x60110+ac*Nf*x00110*x60210+nl*x60300-dc*x60310-dg*x60310-ne*x60310-uptcg*x60310-bbin*Nf*x00011*x60310-ar*Nf*(x01010+x02010)*x60310-ar*Nf*(x01011+x02011)*x60310+ubc*x60311+unbbin*x60311+dr*(x61310+x62310)+dr*(x61311+x62311);
168. x60311_dot = gto*x40311+hto*x50311+agp*Nf*x00210*x60111+ac*Nf*x00110*x60211+bbin*Nf*x00011*x60310-dc*x60311-dg*x60311-ubc*x60311-unbbin*x60311-uptcg*x60311-ar*Nf*(x01010+x02010)*x60311+dr*(x61311+x62311);
169. x61000_dot = ar*x01000*x60000-dr*x61000-nl*x61000-ac*x00100*x61000-agp*x00200*x61000+ne*x61010+dc*x61100+dg*x61200;
170. x61010_dot = ar*Nf*x01010*x60010+nl*x61000-dr*x61010-ne*x61010-bbin*Nf*x00011*x61010-ac*Nf*x00110*x61010-agp*Nf*x00210*x61010+unbbin*x61011+dc*x61110+dg*x61210;
171. x61011_dot = ar*Nf*x01011*x60010+ar*Nf*x01010*x60011+bbin*Nf*x00011*x61010-2*dr*x61011-unbbin*x61011-ac*Nf*x00110*x61011-agp*Nf*x00210*x61011+dc*x61111+dg*x61211;
172. x61100_dot = ar*x01000*x60100+ac*x00100*x61000-dc*x61100-dr*x61100-nl*x61100-agp*x00200*x61100+ne*x61110+dg*x61300;

173. x61110_dot = ar*Nf*x01010*x60110+ac*Nf*x00110*x61010+nl*x61100-dc*x61110-dr*x61110-ne*x61110-bbin*Nf*x00011*x61110-agp*Nf*x00210*x61110+unbbin*x61111+dg*x61310;
174. x61111_dot = ar*Nf*x01011*x60110+ar*Nf*x01010*x60111+ac*Nf*x00110*x61011+bbin*Nf*x00011*x61110-dc*x61111-2*dr*x61111-unbbin*x61111-agp*Nf*x00210*x61111+dg*x61311;
175. x61200_dot = gto*x41200+ar*x01000*x60200+agp*x00200*x61000-dg*x61200-dr*x61200-nl*x61200-ac*x00100*x61200+ne*x61210+dc*x61300;
176. x61210_dot = gto*x41210+ar*Nf*x01010*x60210+agp*Nf*x00210*x61010+nl*x61200-dg*x61210-dr*x61210-ne*x61210-bbin*Nf*x00011*x61210-ac*Nf*x00110*x61210+unbbin*x61211+dc*x61310;
177. x61211_dot = gto*x41211+ar*Nf*x01011*x60210+ar*Nf*x01010*x60211+agp*Nf*x00210*x61011+bbin*Nf*x00011*x61210-dg*x61211-2*dr*x61211-unbbin*x61211-ac*Nf*x00110*x61211+dc*x61311;
178. x61300_dot = gto*x41300+ar*x01000*x60300+agp*x00200*x61100+ac*x00100*x61200-dc*x61300-dg*x61300-dr*x61300-nl*x61300+ne*x61310;
179. x61310_dot = gto*x41310+ar*Nf*x01010*x60310+agp*Nf*x00210*x61110+ac*Nf*x00110*x61210+nl*x61300-dc*x61310-dg*x61310-dr*x61310-ne*x61310-bbin*Nf*x00011*x61310+unbbin*x61311;
180. x61311_dot = gto*x41311+ar*Nf*x01011*x60310+ar*Nf*x01010*x60311+agp*Nf*x00210*x61111+ac*Nf*x00110*x61211+bbin*Nf*x00011*x61310-dc*x61311-dg*x61311-2*dr*x61311-unbbin*x61311;
181. x62000_dot = ar*x02000*x60000-dr*x62000-nl*x62000-ac*x00100*x62000-agp*x00200*x62000+ne*x62010+dc*x62100+dg*x62200;
182. x62010_dot = ar*Nf*x02010*x60010+nl*x62000-dr*x62010-ne*x62010-bbin*Nf*x00011*x62010-ac*Nf*x00110*x62010-agp*Nf*x00210*x62010+unbbin*x62011+dc*x62110+dg*x62210;
183. x62011_dot = ar*Nf*x02011*x60010+ar*Nf*x02010*x60011+bbin*Nf*x00011*x62010-2*dr*x62011-unbbin*x62011-ac*Nf*x00110*x62011-agp*Nf*x00210*x62011+dc*x62111+dg*x62211;
184. x62100_dot = ar*x02000*x60100+ac*x00100*x62000-dc*x62100-dr*x62100-nl*x62100-agp*x00200*x62100+ne*x62110+dg*x62300;
185. x62110_dot = ar*Nf*x02010*x60110+ac*Nf*x00110*x62010+nl*x62100-dc*x62110-dr*x62110-ne*x62110-bbin*Nf*x00011*x62110-agp*Nf*x00210*x62110+unbbin*x62111+dg*x62310;
186. x62111_dot = ar*Nf*x02011*x60110+ar*Nf*x02010*x60111+ac*Nf*x00110*x62011+bbin*Nf*x00011*x62110-dc*x62111-2*dr*x62111-unbbin*x62111-agp*Nf*x00210*x62111+dg*x62311;
187. x62200_dot = gto*x42200+ar*x02000*x60200+agp*x00200*x62000-dg*x62200-dr*x62200-nl*x62200-ac*x00100*x62200+ne*x62210+dc*x62300;
188. x62210_dot = gto*x42210+ar*Nf*x02010*x60210+agp*Nf*x00210*x62010+nl*x62200-dg*x62210-dr*x62210-ne*x62210-bbin*Nf*x00011*x62210-ac*Nf*x00110*x62210+unbbin*x62211+dc*x62310;
189. x62211_dot = gto*x42211+ar*Nf*x02011*x60210+ar*Nf*x02010*x60211+agp*Nf*x00210*x62011+bbin*Nf*x00011*x62210-dg*x62211-2*dr*x62211-unbbin*x62211-ac*Nf*x00110*x62211+dc*x62311;
190. x62300_dot = gto*x42300+ar*x02000*x60300+agp*x00200*x62100+ac*x00100*x62200-dc*x62300-dg*x62300-dr*x62300-nl*x62300+ne*x62310;
191. x62310_dot = gto*x42310+ar*Nf*x02010*x60310+agp*Nf*x00210*x62110+ac*Nf*x00110*x62210+nl*x62300-dc*x62310-dg*x62310-dr*x62310-ne*x62310-bbin*Nf*x00011*x62310+unbbin*x62311;
192. x62311_dot = gto*x42311+ar*Nf*x02011*x60310+ar*Nf*x02010*x60311+agp*Nf*x00210*x62111+ac*Nf*x00110*x62211+bbin*Nf*x00011*x62310-dc*x62311-dg*x62311-2*dr*x62311-unbbin*x62311;
*193. x70000_dot = tlph*McPh-upuh*x70000-an*x70000*x10000+dn*x80000;*
*194. x80000_dot = an*x70000*x10000-dn*x80000-acph*x00100*x80000+dc*x80100-upuoh*x80000;*
*195. x80100_dot = acph*x00100*x80000-dc*x80100-hoho*x80100-upuoh*x80100;*
*196. x90000_dot = -acph*x90000*x00100+dc*x90100-upoh*x90000+an*xa0000*x20000-dn*x90000-nl*x90000+ne*x90010;*
*197. x90010_dot = unbbin*x90011-bbin*Nf*x00011*x90010+ubc*x90011-upoh*x90010+nl*x90000-ne*x90010+an*Nf*x20010*xa0010-dn*x90010-acph*Nf*x00110*x90010+dc*x90110;*
*198. x90011_dot = -upoh*x90011-ubc*x90011+bbin*Nf*x00011*x90010-unbbin*x90011-acph*Nf*x90011*x00110+dc*x90111+an*Nf*x20010*xa0011-dn*x90011*2+an*Nf*x20011*xa0010;*
*199. x90100_dot = hoho*x80100+acph*x00100*x90000-dc*x90100-nl*x90100-upoh*x90100+ne*x90110;*
*200. x90110_dot = -bbin*Nf*x90110*x00011+unbbin*x90111+ubc*x90111+nl*x90100-ne*x90110+acph*Nf*x90010*x00110-dc*x90110-upoh*x90110;*
*201. x90111_dot = acph*Nf*x00110*x90011-dc*x90111-ubc*x90111+bbin*Nf*x90110*x00011-unbbin*x90111-upoh*x90111;*
*202. xa0000_dot = -an*xa0000*x20000+dn*x90000-uph*xa0000-nl*xa0000+ne*xa0010;*
*203. xa0010_dot = -an*Nf*x20010*xa0010+dn*x90010-an*Nf*x20011*xa0010+dn*x90011-uph*xa0010+nl*xa0000-ne*xa0010-arph*Nf*xa0010*(x01010+x02010)+drph*(xa1010+xa2010)-bbin*Nf*xa0010*x00011+ubc*xa0011+unbbin*xa0011-ar*Nf*xa0010*(x01011+x02011)+dr*(xa1011+xa2011);*
*204. xa0011_dot = bbin*Nf*xa0010*x00011-ubc*xa0011-unbbin*xa0011-uph*xa0011-arph*Nf*(x01010+x02010)*xa0011+drph*(xa1011+xa2011)-an*xa0011*Nf*x20010+dn*x90011;*
*205. xa1010_dot = arph*Nf*xa0010*x01010-drph*xa1010-bbin*Nf*x00011*xa1010+unbbin*xa1011;*
*206. xa1011_dot = bbin*Nf*x00011*xa1010-unbbin*xa1011+arph*Nf*xa0011*x01010+arph*Nf*xa0010*x01011-drph*2*xa1011;*
*207. xa2010_dot = arph*Nf*xa0010*x02010-drph*xa2010-bbin*Nf*xa2010*x00011+unbbin*xa2011;*
*208. xa2011_dot = bbin*Nf*x00011*xa2010-unbbin*xa2011+arph*Nf*xa0011*x02010+arph*Nf*xa0010*x02011-drph*2*xa2011;*

Table S5. Model knockout conditions. "Short" represents period shortening, "long" represents period lengthening, "AR" represents arrhythmicity, "KD" represents knockdown, and "WT" represents a wild-type phenotype. When available, amount of increase or decrease in period length is specified. Sustained oscillations are represented by the following: a minimum of 3 peaks, an average period between 23.8 and 24.6, a midpoint peak:trough ratio greater than or equal to 1.5, an endpoint peak:trough ratio greater than or equal to 1.5, and a ratio of the midpoint peak:trough ratio to the endpoint peak:trough ratio of less than or equal to 1.2.

Knockdown	Effects	Model Representation
PER1$^{-/-}$	• WT *PER2*[19]<br>• Short or AR *BMAL1*[18]	Nuclear *Per2* mRNA:<br>o Sustained oscillations<br>o Period after KD is within 1% of WT period<br>o Amplitude after KD is within 20% of WT period<br>Nuclear *Bmal1* mRNA:<br>o At least 3 peaks<br>o Period after KD between 94 and 98% of WT period<br>OR<br>o Ratio of midpoint peak:trough ratio to final peak:trough ratio > 1.5
PER2$^{-/-}$	• Short *BMAL1*[18]	Nuclear *Bmal1* mRNA:<br>o At least 3 peaks<br>o Period after KD between 94 and 98% of WT period
PER3$^{-/-}$	• Short (-0.5 h) *PER2*[19]<br>• Short (-0.62 h) *BMAL1*[17]	Nuclear *Per2* mRNA:<br>o At least 3 peaks<br>o Period after KD between 0 and 0.8 h shorter than WT period<br>Nuclear *Bmal1* mRNA:<br>o At least 3 peaks<br>o Period after KD between 94 and 98% of WT period
CRY1$^{-/-}$	• Short (-1.88 h) *PER2*[19]<br>• Short *BMAL1*[17]	Nuclear *Per2* mRNA:<br>o At least 3 peaks<br>o Period after KD between 1.2 and 3 h shorter than WT period<br>Nuclear *Bmal1* mRNA:<br>o At least 3 peaks<br>o Period after KD between 94 and 98% of WT period
CRY2$^{-/-}$	• Long (+1.62 h) PER2[19]<br>• Long *BMAL1*[17]	Nuclear *Per2* mRNA:<br>o At least 3 peaks<br>o Period after KD between 1.2 and 3 h longer than WT period<br>Nuclear *Bmal1* mRNA:<br>o At least 3 peaks<br>o Period after KD more than 2% greater than WT period, but not more than 3 h greater
CRY1$^{-/-}$/***CRY2***$^{-/-}$	• AR *BMAL1*[17]	Nuclear *Bmal1* mRNA:<br>o Ratio of midpoint peak:trough ratio to endpoint peak:trough ratio > 1.5
BMAL1$^{-/-}$	• AR *PER2*[13] or short *PER2*[18]	Nuclear *Per2* mRNA:<br>o Ratio of midpoint peak:trough ratio to endpoint peak:trough ratio > 1.5<br>OR<br>o At least 3 peaks<br>o Period after KD between 94 and 98% of WT period
NPAS2$^{-/-}$	• WT *BMAL1*[17]	Nuclear *Bmal1* mRNA:<br>o Sustained oscillations<br>o Period after KD is within 1% of WT period<br>o Amplitude after KD is within 20% of WT period

Table S6. Conditions for SNPs and VNTRs.

	Proposed Molecular Effects	Model Representation of Effects	Observed phenotype	Model Representation of Phenotype
**PER3-P415A/H417R**[9]	• Decreased protein stability<br>• Reduced PER3 binding to other PER proteins	• Increased PER3 degradation rates<br>• Decreased PER1-PER3 binding rate	Association with FASP	Average period of *Bmal1 mRNA* decreases by 2-6% from WT
***PER3*[4] homozygotes**[7]	Decreased phosphorylation compared to *PER3*[45] heterozygotes	Decrease PER3 phosphorylation.	Associated with extreme evening preference or DSP	Average period of *Bmal1 mRNA* increases by 2-6% from WT
***PER3*[5] homozygotes**[7]	Increased phosphorylation compared to *PER3*[45] heterozygotes	Increase PER3 phosphorylation	Associated with morning preference.	Average period of *Bmal1 mRNA* decreases by 2-6% from WT
**PER3-P864A**[8]	Mutation in CKI binding domain leading to decreased CKI-mediated phosphorylation	Decrease PER3-CKI binding	Associated with eveningness or FRT	Average period of *Bmal1 mRNA* increases by 2-6% from WT or gradually lengthening period in *Bmal1 mRNA*

Table S7. Results of SNP/VNTR modeling conditions. We chose 1,000 parameter sets that satisfied all experimental knockout conditions, and we tested each set with conditions to represent each SNP or VNTR. This procedure was repeated a total of three times, and the average number of sets passing the conditions (±2 SE) is reported for each trial. All SNP and VNTR conditions are explained in Table S6.

**Polymorphism/Parameter Variation**	**20% decrease**	**40% decrease**	**60% decrease**	**80% decrease**	**100% decrease**
**PER3-P415A/H417R:** 4-fold increase in PER3 degradation rate, decreased PER1-PER3 binding	194±32	268±41	362±39	500±29	642±26
**PER3-P864A:** decreased PER3-CKI binding	42+12	43±12	42±12	34±10	0±0
***PER3*[4] homozygotes:** decreased PER3 phosphorylation rate	114±20	270±25	526±40	723±29	612±29
	**4-fold increase**	**8-fold increase**	**12-fold increase**	**16-fold increase**	**20-fold increase**
**PER3-P415A/H417R:** increased PER3 degradation rate, with 20% decreased PER1-PER3 binding	194±32	436±25	530±23	577±25	603±29
***PER3*[5] homozygotes:** increased PER3 phosphorylation rate	612±29	723±29	526±39	270±25	114±20

References

1. Kerkhof, G. A. & Van Dongen, H. P. A. Morning-type and evening-type individuals differ in the phase position of their endogenous circadian oscillator. *Neurosci. Lett.* **218,** 153–156 (1996).
2. Taillard, J., Philip, P., Coste, O., Sagaspe, P. & Bioulac, B. The circadian and homeostatic modulation of sleep pressure during wakefulness differs between morning and evening chronotypes. *J. Sleep Res.* **12,** 275–282 (2003).
3. Roepke, S. E. & Duffy, J. F. Differential impact of chronotype on weekday and weekend sleep timing and duration. *Nat. Sci. Sleep* **2,** 213–220 (2010).
4. Spielberger, C. D., Gorsuch, R. L., Lushene, R., Vagg, P. R., & Jacobs, G. A. *Manual for the State-Trait Anxiety Inventory*. (Consulting Psychologists Press, 1983).
5. Ebisawa, T. *et al.* Association of structural polymorphisms in the human period3 gene with delayed sleep phase syndrome. *EMBO Rep.* **2,** 342–346 (2001).
6. Barclay, N. L. *et al.* Sleep quality and diurnal preference in a sample of young adults: Associations with 5HTTLPR, PER3, and CLOCK 3111. *Am. J. Med. Genet. Part B Neuropsychiatr. Genet.* **156,** 681–690 (2011).
7. Archer, S. N. *et al.* A length polymorphism in the circadian clock gene Per3 is linked to delayed sleep phase syndrome and extreme diurnal preference. *Sleep* **26,** 413–415 (2003).
8. Hida, A. *et al.* Screening of clock gene polymorphisms demonstrates association of a PER3 polymorphism with morningness-eveningness preference and circadian rhythm sleep disorder. *Sci. Rep.* **4,** 6309 (2014).
9. Zhang, L. *et al.* A PERIOD3 variant causes a circadian phenotype and is associated with a seasonal mood trait. *Proc. Natl. Acad. Sci. U. S. A.* **113,** E1536–E1544 (2016).
10. Gustafson, C. & Partch, C. Emerging models for the molecular basis of mammalian circadian timing. *Biochemistry* (2014).
11. Lee, C., Weaver, D. R. & Reppert, S. M. Direct Association between Mouse PERIOD and CKIε Is Critical for a Functioning Circadian Clock. *Mol. Cell. Biol.* **24,** 584–594 (2004).
12. Jolley, C. C., Ukai-Tadenuma, M., Perrin, D. & Ueda, H. R. A mammalian circadian clock model incorporating daytime expression elements. *Biophys. J.* **107,** 1462–1473 (2014).
13. Kim, J. K. & Forger, D. B. A mechanism for robust circadian timekeeping via stoichiometric balance. *Mol. Syst. Biol.* **8,** 630 (2012).
14. Ogawa, Y. *et al.* Positive autoregulation delays the expression phase of mammalian clock gene per2. *PLoS One* **6,** (2011).
15. Relógio, A. *et al.* Tuning the mammalian circadian clock: Robust synergy of two loops. *PLoS Comput. Biol.* **7,** e1002309 (2011).
16. Mirsky, H. P., Liu, A. C., Welsh, D. K., Kay, S. A. & Doyle, F. J. A model of the cell-autonomous mammalian circadian clock. *Proc. Natl. Acad. Sci. U. S. A.* **106,** 11107–11112 (2009).
17. Baggs, J. E. *et al.* Network features of the mammalian circadian clock. *PLoS Biol.* **7,** 0563–0575 (2009).
18. Ramanathan, C. *et al.* Cell Type-Specific Functions of Period Genes Revealed by Novel Adipocyte and Hepatocyte Circadian Clock Models. *PLoS Genet.* **10,** (2014).
19. Liu, A. C. *et al.* Intercellular Coupling Confers Robustness against Mutations in the SCN Circadian Clock Network. *Cell* **129,** 605–616 (2007).
